# The human AAA-ATPase VPS4A isoform and its co-factor VTA1 have a unique function in regulating mammalian cytokinesis abscission

**DOI:** 10.1371/journal.pbio.3002327

**Published:** 2024-04-30

**Authors:** Inbar Dvilansky, Yarin Altaras, Nikita Kamenetsky, Dikla Nachmias, Natalie Elia

**Affiliations:** 1 Department of Life Sciences, Ben-Gurion University of the Negev, Beer Sheva, Israel; 2 National Institute for Biotechnology in the Negev (NIBN), Ben-Gurion University of the Negev, Beer Sheva, Israel; Utrecht University, NETHERLANDS

## Abstract

Mutations in the human AAA-ATPase VPS4 isoform, VPS4A, cause severe neurodevelopmental defects and congenital dyserythropoietic anemia (CDA). VPS4 is a crucial component of the endosomal sorting complex required for transport (ESCRT) system, which drives membrane remodeling in numerous cellular processes, including receptor degradation, cell division, and neural pruning. Notably, while most organisms encode for a single VPS4 gene, human cells have 2 VPS4 paralogs, namely VPS4A and VPS4B, but the functional differences between these paralogs is mostly unknown. Here, we set out to investigate the role of the human VPS4 paralogs in cytokinetic abscission using a series of knockout cell lines. We found that VPS4A and VPS4B hold both overlapping and distinct roles in abscission. VPS4A depletion resulted in a more severe abscission delay than VPS4B and was found to be involved in earlier stages of abscission. Moreover, VPS4A and a monomeric-locked VPS4A mutant bound the abscission checkpoint proteins CHMP4C and ANCHR, while VPS4B did not, indicating a regulatory role for the VPS4A isoform in abscission. Depletion of VTA1, a co-factor of VPS4, disrupted VPS4A-ANCHR interactions and accelerated abscission, suggesting that VTA1 is also involved in the abscission regulation. Our findings reveal a dual role for VPS4A in abscission, one that is canonical and can be compensated by VPS4B, and another that is regulatory and may be delivered by its monomeric form. These observations provide a potential mechanistic explanation for the neurodevelopmental defects and other related disorders reported in VPS4A-mutated patients with a fully functional VPS4B paralog.

## Abstract 2

The human AAA-ATPase VPS4 facilitates the complete separation between daughter cells at the end of cytokinesis. It has two paralogs, VPS4A and VPS4B, but whether there are functional differences between them is unknown. This study shows that VPS4A has unique regulatory functions in cytokinesis for which VPS4B cannot compensate, explaining the neurodevelopmental defects recently reported in people with mutations in VPS4A.

## Introduction

VPS4 is one of the core components of the endosomal sorting complex required for transport (ESCRT) membrane remodeling system, which is currently recognized as one of the most basic cellular machineries for driving membrane fission in cells. As such, VPS4 is essential for in vitro reconstitution of ESCRT-induced membrane fission and is involved in all canonical ESCRT-mediated processes in eukaryotes, including in multivesicular body (MVB) formation, release of retroviruses from the cell surface, nuclear envelope sealing, plasma membrane repair, neural pruning, and scission of daughter cells during the last stages of cytokinesis [1–3]. Additional non-canonical roles for VPS4 in centrosomes have also been reported [4,5]. Finally, mutations in VPS4 genes were associated with different neuro-pathologies and cancer [6–9]. Therefore, understanding the basis for VPS4 function in cells is of great interest.

VPS4 is an AAA-ATPase that assembles into functional hexamers or dodecamers that hydrolyze ATP [10,11]. This functional hexamer is stabilized by binding to its co-factor VTA1, which dimerizes to form a bridge that connects 2 adjacent VPS4 subunits [11,12]. While yeast cells encode for a single VPS4 protein, mammalian cells encode for 2 VPS4 homologs, namely VPS4A and VPS4B. Given the high sequence similarity of these paralogs (81% amino acids identity), they were traditionally thought to have redundant functions in cells [8,13]. However, recent evidence suggests that the cellular functions of VPS4A and VPS4B do not fully overlap. First, mutations in VPS4A in patients carrying normal VPS4B caused severe diseases associated with structural brain abnormalities, neurodevelopmental defects, cataract, growth defects, and congenital dyserythropoietic anemia (CDA) [6,7]. Second, while VPS4A was reported to function as a tumor suppressor, VPS4B exhibited pro- or anti-oncogenic activities in different cancers [8,9]. Nevertheless, depleting VPS4A in cancer cells with loss of VPS4B led to synthetic cell death, suggesting at least partial overlap between these isoforms [8]. Third, VPSB was previously shown to be more crucial for HIV viral release than VPS4A, and we recently substantiated these findings and showed that depletion of VPS4A or VPS4B has differential effects on HIV-viral release, suggesting a unique property for each isoform [14,15]. Collectively, these reports raise the possibility that the 2 VPS4 isoforms hold both overlapping and unique functions in cells stressing the need to investigate the specific function differences of each isoform in cells.

Cytokinetic abscission provides an excellent platform for dissecting the role of VPS4 paralogs. During abscission, components of the ESCRT-III complex assemble into helical filaments at the intercellular bridge that connect the 2 dividing daughter cells, and drive constriction and fission of the membranes connecting these cells to complete the division process. VPS4 participates in several crucial aspects of this process. First, it is involved in the exchange of ESCRT-III monomers within the filament during filament assembly [16]. Second, it plays a role in ESCRT-III depolymerization and membrane fission that terminates abscission [17,18]. Third, it interacts with proteins of the AuroraB abscission checkpoint (ANCHR and CHMP4C), which regulates abscission timing [19,20]. Notably, both paralogs were shown to localize at the intercellular bridge when overexpressed in cells [16,18], suggesting their functional involvement in abscission. Therefore, VPS4 exhibits multiple functions in abscission, with both VPS4 paralogs potentially contributing to these functions.

Here, we set out to investigate the functions of the 2 VPS4 isoforms encoded in mammalian cells during cytokinetic abscission by knocking out specific VPS4 components. We found that although cells express higher levels of VPS4B compared to VPS4A, depletion of VPS4A leads to a considerably more severe abscission delay than depletion of VPS4B. Stochastic optical reconstruction microscopy (STORM) measurements of the density of the ESCRT-III protein, IST1, at the intracellular bridge pointed to the involvement of VPS4A in early abscission and of VPS4B in late abscission, supporting a differential role for VPS4 isoforms. Unexpectedly, depletion of the VPS4 co-factor, VTA1, which stabilizes the active hexameric form of both VPS4 isoforms, accelerated abscission. Moreover, in WT cells, VTA1 was found to interact with both CHMP4C and ANCHR, suggesting its role in the abscission checkpoint. VPS4A and a VPS4A mutant defected in hexamerization (mVPS4A), but not VPS4B, were also found to interact with CHMP4C and ANCHR. However, VPS4A-ANCHR interactions were abolished in VTA1 KO cells, suggesting the formation of a VPS4A-VTA1-ANCHR complex at the abscission checkpoint. Collectively, our data indicate that VPS4 A and B isoforms have distinct functions remodeling the ESCRT-III filament during cytokinetic abscission that could be partially compensated by the presence of one isoform. However, the regulatory role of VPS4 in abscission is mediated by the VPS4A isoform, potentially in its monomeric form, and is dependent on VTA1 binding. Notably, the regulatory role of VPS4A shown here provide for the first time a mechanistic explanation for the previously reported direct involvement of VPS4A (but not VPS4B) in disease development [6,7].

## Results

**Fig 1:**
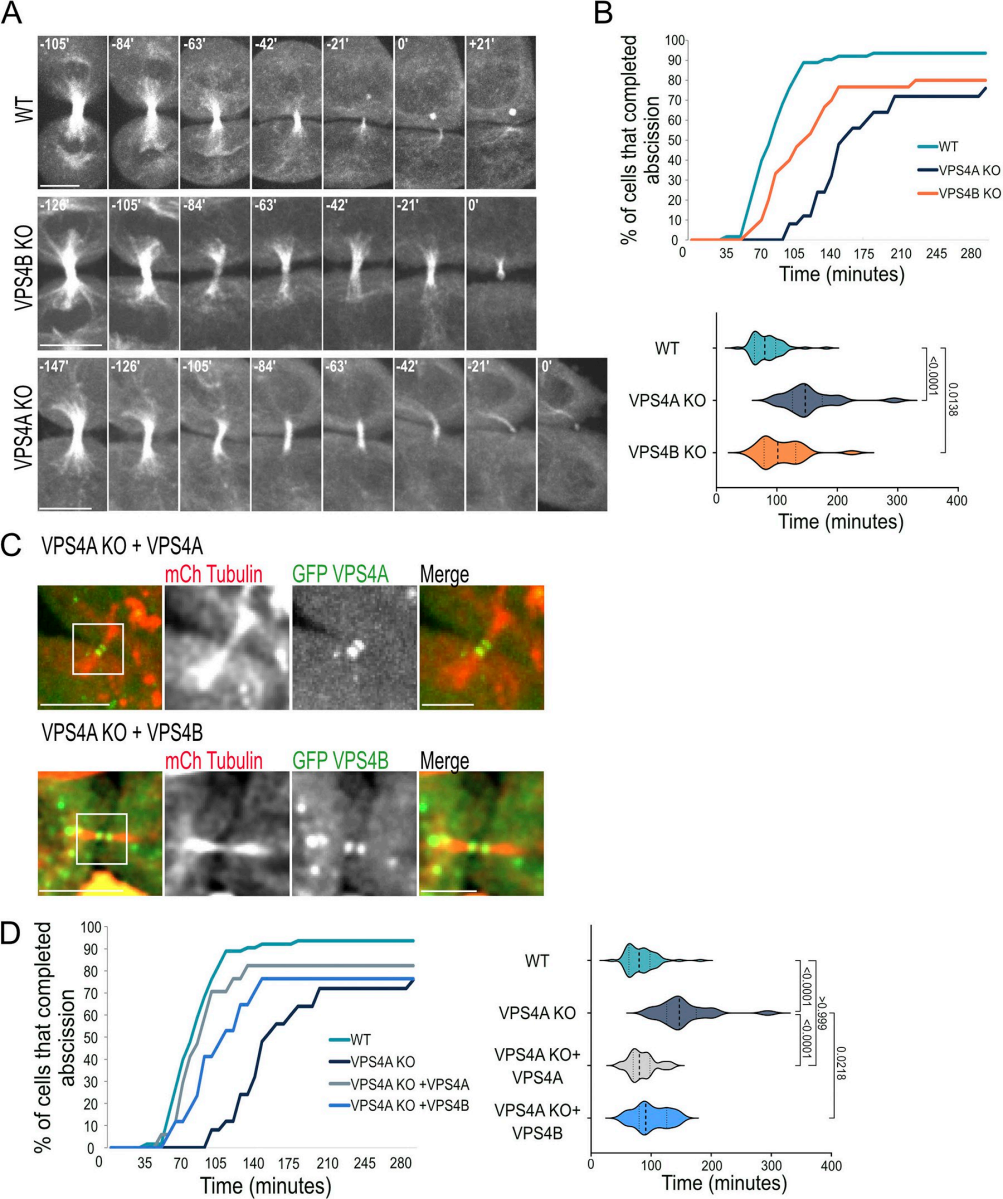
Knock-out of VPS4A causes a stronger abscission delay phenotype than VPS4B knock-out. (**A**) Live cell imaging of WT (top panel), VPS4B KO (middle panel), and VPS4A KO (bottom panel), transfected with GFP-tubulin. Z slices were captured at 7-min intervals using a confocal spinning-disk microscope. Maximum-intensity projections of representative cells at various time points during cytokinesis are shown. The abscission time was set as time = 0 and was defined as the time of the first cleavage event of the microtubule bridge. Scale, 10 μm. (**B**) Duration of abscission (from cleavage furrow formation to microtubule bridge cleavage) as measured for WT, VPS4A KO and VPS4B KO cells. Top panel, a cumulative plot; bottom panel, a violin plot showing the distribution of abscission times. Averaged abscission times: VPS4A KO 154 ± 45 min, *n* = 25; VPS4B KO 107 ± 37 min, *n* = 30; WT 83 ± 25 min, *n* = 63. Data for each condition were obtained from at least 3 independent experiments (S1 Data), and *p* values were calculated using Anova (Kruskal–Wallis test). (**C**) The abscission delay observed in VPS4A KO cells is fully rescued by exogenous expression of VPS4A and partially rescued by overexpression of VPS4B. VPS4A KO cells were co-transfected with mCherry-tubulin (red) together with either GFP-VPS4A (top panel, green) or GFP-VPS4B (bottom panel, green), and imaged 48 h later, at 7-min intervals. Left panels: zoom-out images (scale, 10 μm). Zoom-in images of the white squares in left panels are shown on the right (scale, 5 μm). Shown are maximum-intensity projection images of representative cells. Note the arrival of VPS4A and VPS4B to the intercellular bridge in VPS4A KO cells. Complete movie series are provided in S1 Movie (top panel) and S2 Movie (bottom panel). (**D**) Duration of abscission (from cleavage furrow formation to microtubule bridge cleavage) as measured for VPS4A KO cells expressing either VPS4A or VPS4B. Left panel, a cumulative plot. Right panel, a violin plot showing the distribution of abscission times. WT and VPS4A KO cell data (reproduced from Fig 1A and 1B) are shown for reference. Averaged abscission times: VPS4A KO + GFP-VPS4A 85 ± 22 min, *n* = 17; VPS4A KO + GFP-VPS4B 100 ± 28 min, *n* = 17. Similar abscission rates were observed in WT cells expressing either GFP-tubulin or mCherry-tubulin (S1C Fig). Data were obtained from 5 independent experiments of each condition (S1 Data), and *p* values were calculated using Anova (Kruskal–Wallis test).

To dissect the role of VPS4 isoforms in cytokinesis, we monitored abscission in VPSA and VPS4B KO cells recently generated in our laboratory (S1A Fig) [14]. Attempts to generate cells depleted of both VPS4A and VPS4B resulted in cell death, suggesting that at least 1 VPS4 isoform is needed for cell viability, as previously suggested [8]. Depleting either VPS4A or VPS4B caused a delay in abscission and an increase in the percentage of cells that failed to complete abscission but did not affect the formation or morphology of the intercellular bridge (Fig 1A and 1B). VPS4A KO cells exhibited a considerably more severe abscission delay (averaged abscission time of 154 min in VPS4A KO versus 107 min in VPS4B KO compared to 83 min in WT cells). No increase in multinucleated cells was observed in VPS4A KO cells, suggesting the overall cytokinesis is not significantly affected (S1B Fig). Exogenously expressed VPS4A arrived at the intercellular bridge and fully rescued the abscission delay phenotype (Figs 1C and 1D, and S1C–S1D). Expressing exogenous VPS4B at similar levels (S1E Fig), only partially rescued the abscission delay (Fig 1C and 1D), indicating that VPS4B cannot fully compensate for the loss of VPS4A in abscission. Moreover, measurements of the total levels of endogenous VPS4 paralogs in WT cells revealed that VPS4B is considerably more abundant than VPS4A (approximately 5:1, respectively), indicating that depletion of VPS4A has smaller effect on the overall levels of VPS4 levels in the cell compared to VPS4B depletion (S1F Fig). Collectively, these findings suggest that, irrespectively to its expression levels, VPS4A is the more prominent paralog in abscission.

**Fig 2:**
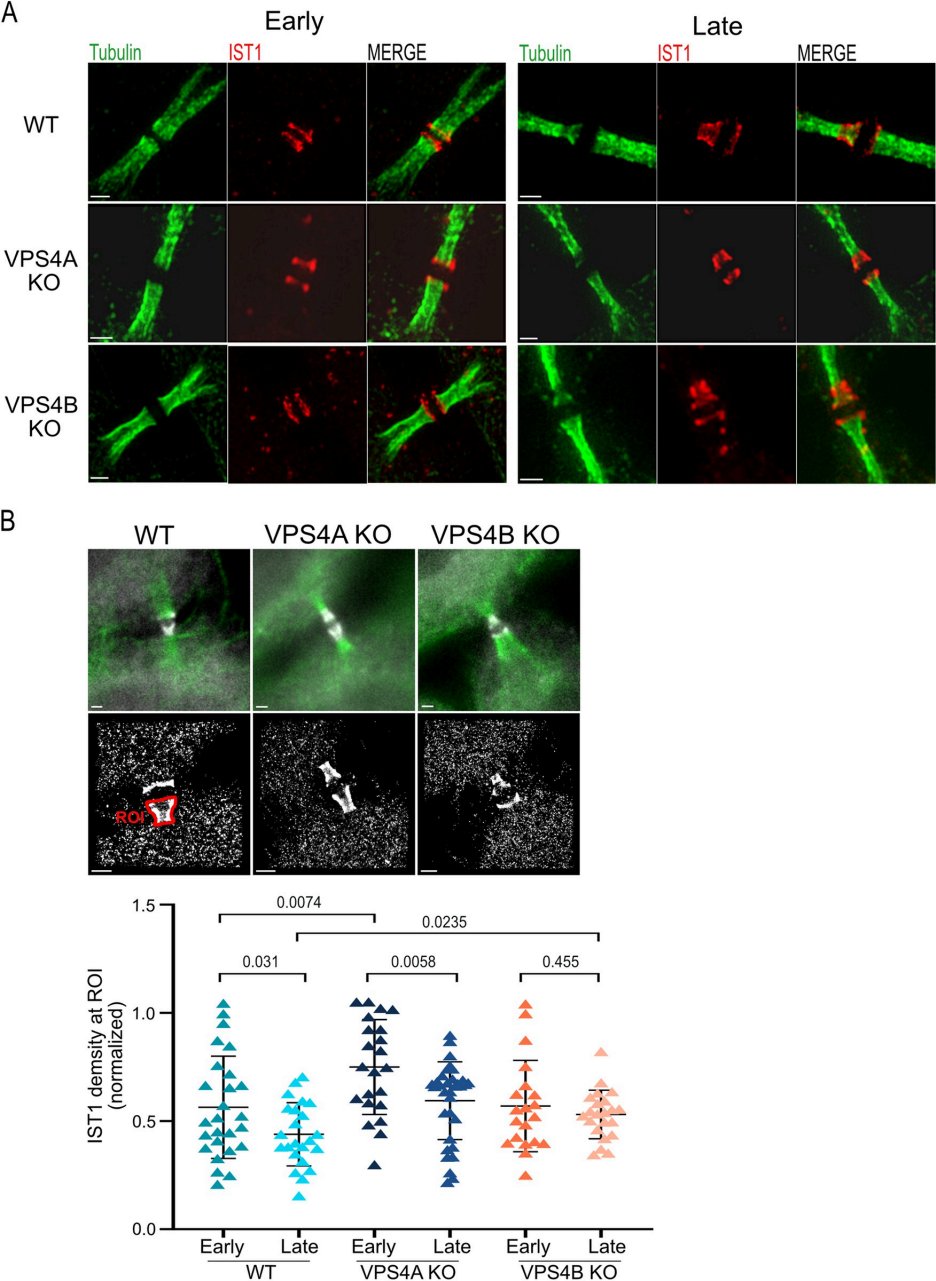
Differential effect of VPS4A and VPS4B depletion on the distribution of ESCRT-III at the intercellular bridge. (**A**) SIM imaging shows normal ESCRT-III organization at the intercellular bridge in VPS4 KO cells. WT (top panel), VPS4A KO (middle panel), and VPS4B KO (bottom panel) cells were stained with anti-α-tubulin (green) and anti-IST1 (red) antibodies and imaged by SIM. Similar results were obtained using anti-CHMP4B (S2A Fig). Shown are maximum projection images of representative cells obtaining early (left) and late (right) intercellular bridges. Data was reproduced in at least 3 independent experiments. Scale, 1 μm. (**B**) STORM analysis shows different densities of the ESCRT-III protein IST1 at intercellular bridges of VPS4A and VPS4B KO cells. Cells were synchronized, stained with anti-α-tubulin and anti-IST1 antibodies, and imaged by 2D STORM in epifluorescence mode (see Methods). Top panel: wide field images showing the intercellular bridge and IST1 staining (tubulin, green; IST1, white); middle panel: the STORM datasets of IST1 localizations obtained for bridges in top panel; bottom panel: a scatter plot graph showing the density of IST1 at the intercellular of all cells measured. IST1 density was calculated by dividing the total number of localizations by the area of manually selected regions (ROIs) that include IST1 staining on one side of the bridge (red marking, middle left panel). Plots showing area and number of localizations measured for each ROI at the different conditions are presented in S2B Fig. Calculations were performed on data from 5 independent experimental repeats, and *p* values were calculated using unpaired *t* test. WT: early, *n* = 26, late, *n* = 23; VPSA KO: early, *n* = 22, late, *n* = 33; VPSB KO: early, *n* = 20, late, *n* = 22 (S2 Data). *n* refers to the number of ROIs. ESCRT, endosomal sorting complex required for transport; SIM, structured illumination microscopy; STORM, stochastic optical reconstruction microscopy.

During cytokinetic abscission, components of the ESCRT-III complex were shown to assemble into specific high-ordered structures of rings and spiral at the intracellular bridge at different stages of abscission [18,21,22]. Structured illumination microscopy (SIM) imaging of the ESCRT-III proteins IST1 and CHMP4B in VPS4 KO cells revealed that the overall organization of the ESCRT-III filaments at the intercellular bridge is not perturbed upon depletion of either isoform (Figs 2A and S2A). The typical ring organization could be readily detected in early bridges of cells depleted of VPS4A or VPS4B and elongation of the ESCRT-III polymer toward the cell body was observed in late bridges in both KO cells, supporting proper spiral formation. We therefore concluded that a single VPS4 isoform is sufficient for proper ESCRT-III organization at the intercellular bridge.

As VPS4 was previously shown to mediate the exchange of proteins within the ESCRT-III polymer, we next set to examine the distribution of ESCRT-III proteins at the intercellular bridge in WT and KO cells [16,23]. To this end, we established a STORM-based assay that allowed to quantify the distribution of endogenous ESCRT-III proteins at the intercellular bridge at the single molecule level (see Methods section). Measurements of the number of localization and the total area that contain IST1 signal at the intercellular bridge revealed an increase in the transition from early to late bridges, in both WT and KO cells, supporting the proper ESCRT-III organization obtained by SIM (S2B Fig). Yet, calculations of the density of IST1 at the intercellular bridge pointed to a differential role of VPS4 paralogs in abscission. In WT cells, a decrease in IST1 density was observed in the transition from early to late intercellular bridges, indicating removal of IST1 proteins from the ESCRT-III structure (Fig 2B). While this phenomenon was recapitulated in VPS4A KO cells, it was almost completely abolished in VPS4B KO cells, suggesting that the decrease in IST1 density in late intercellular bridges is driven by the VPS4B isoform. Comparison of the initial IST1 density in early intercellular bridges of WT and KO cells showed that in the absence of VPS4A, but not of VPS4B, IST density at early intercellular bridges is significantly increased, pointing to the involvement of VPS4A in early stages of abscission (Fig 2B). Collectively, these results suggest that both VPS4 isoforms are involved in IST1 dynamics in the ESCRT-III structure. However, while the VPS4A isoform is primarily involved in early stages the VPS4B isoform act in late stages. Compromising either of these properties does not appear to affect the overall organization of the ESCRT-III polymer but may contribute to the delayed abscission phenotype observed in these KO cells. Therefore, VPS4A and VPS4B appear to have temporally separated functions in abscission.

**Fig 3:**
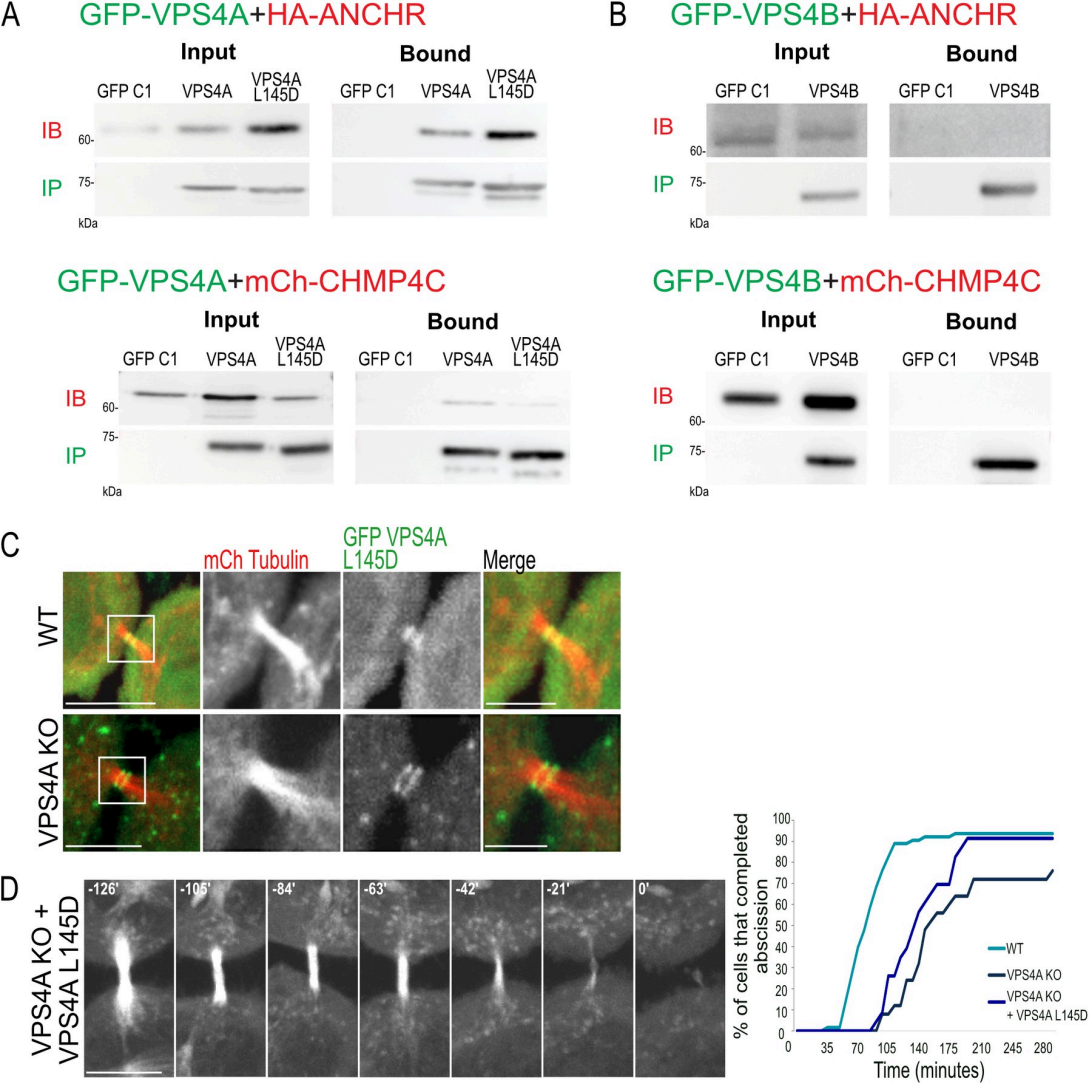
VPS4A is the paralog involved in the abscission checkpoint. (**A, B**) WT cells co-transfected with either GFP-VPS4A, GFP- VPS4A L145D (mVPS4A) (A), or GFP-VPS4B (B) together with HA-ANCHR (top panels) or mCherry-CHMP4C (bottom panels) were subjected to IP using anti-GFP beads. Note that VPS4A and mVPS4A mutant bound the abscission checkpoint proteins, whereas VPS4B does not. Immunoblotting: IB fractions - anti-HA (top panels) or anti-mCherry (bottom panels); IP fractions - anti-GFP. Data were reproduced in at least 2 independent experiments. Complete blots are provided in S1 Raw Images. (**C**) mVPS4A (VPA4A L145D) localizes to the intracellular bridge in WT (top panel) and VPS4A KO (bottom panel) cells. Shown are intercellular bridges of cells that were co-transfected with mCherry-tubulin (red) and GFP-mVPS4A (green). Left panels: zoom-out (scale, 10 μm) images. Zoom-in images of the white squares in left panels are shown on the right (scale, 5 μm). Shown are maximum-intensity projection images of representative cells. Data was reproduced in at least 3 independent experiments. (**D**) Kinetics of abscission in VPS4A KO cells expressing the mVPS4A mutant. Left panel: maximum-intensity projection time-lapse images of dividing VPS4A KO cells co-transfected with mCherry-tubulin and GFP-mVPS4A (scale, 10 μm). The abscission time, which was defined as the time of the first microtubule bridge cleavage event, was set as time 0. Right panel: a cumulative plot of abscission duration (from cleavage furrow formation to microtubule bridge cleavage) of VPS4A KO expressing mVPS4A (averaged time 138 ± 33 min, S3B Fig). Data was obtained from 2 independent experiments, *n* = 23 cells (S1 Data). WT and VPS4A KO cell data (reproduced from Fig 1A and 1B) are shown for reference. IP, immunoprecipitation.

Previous studies indicated a role for VPS4 in the AuroraB abscission checkpoint [19,20,24]. We therefore asked which of the VPS4 isoforms is involved in this checkpoint. In IP experiments, VPS4A, but not VPS4B, bound the abscission checkpoint proteins ANCHR and CHMP4C (Fig 3A and 3B), indicating that the regulatory role, previously reported for VPS4 in abscission, is mediated by VPS4A. In cells, VPS4 resides in a monomeric or dimeric forms at the cytosol and assemble into active hexamers upon binding to membrane-bound ESCRT-III [25]. To examine whether the regulatory role of VPS4A in abscission relies on hexamerization, we generated a VPS4A monomeric mutant based on a previously reported yeast monomeric VPS4 mutant (mVPS4A) (L145D, corresponds to L151D in yeast VPS4, see S3A Fig) [26]. We found, that similar to WT VPS4A, the mVPS4A mutant bind the AuroraB checkpoint proteins ANCHR and CHMP4C (Fig 3A). Moreover, a fluorescently tagged mVPS4A mutant localized at the intercellular bridge in both WT and VPS4A KO cells, indicating that VPS4A can be targeted to the intracellular bridge in its monomeric form (Fig 3C). Expression of mVPS4A in VPS4A KO cells also resulted a slight increase in abscission rate and a complete recovery in the percentages of cells that completed abscission (72% in VPS4A KO versus 91% in mVPS4A OE and 94% in WT cells) (Figs 3D and S3B). These data indicate that the regulatory role of VPS4 in abscission is facilitated by the VPS4A isoform and may be executed by its monomeric form. It, therefore, appears that VPS4A has a dual function in abscission: one that is regulatory and can be mediated by its monomeric form and another that is associated with its canonical function as an AAA-ATPase that remodels the ESCRT-III filament.

**Fig 4:**
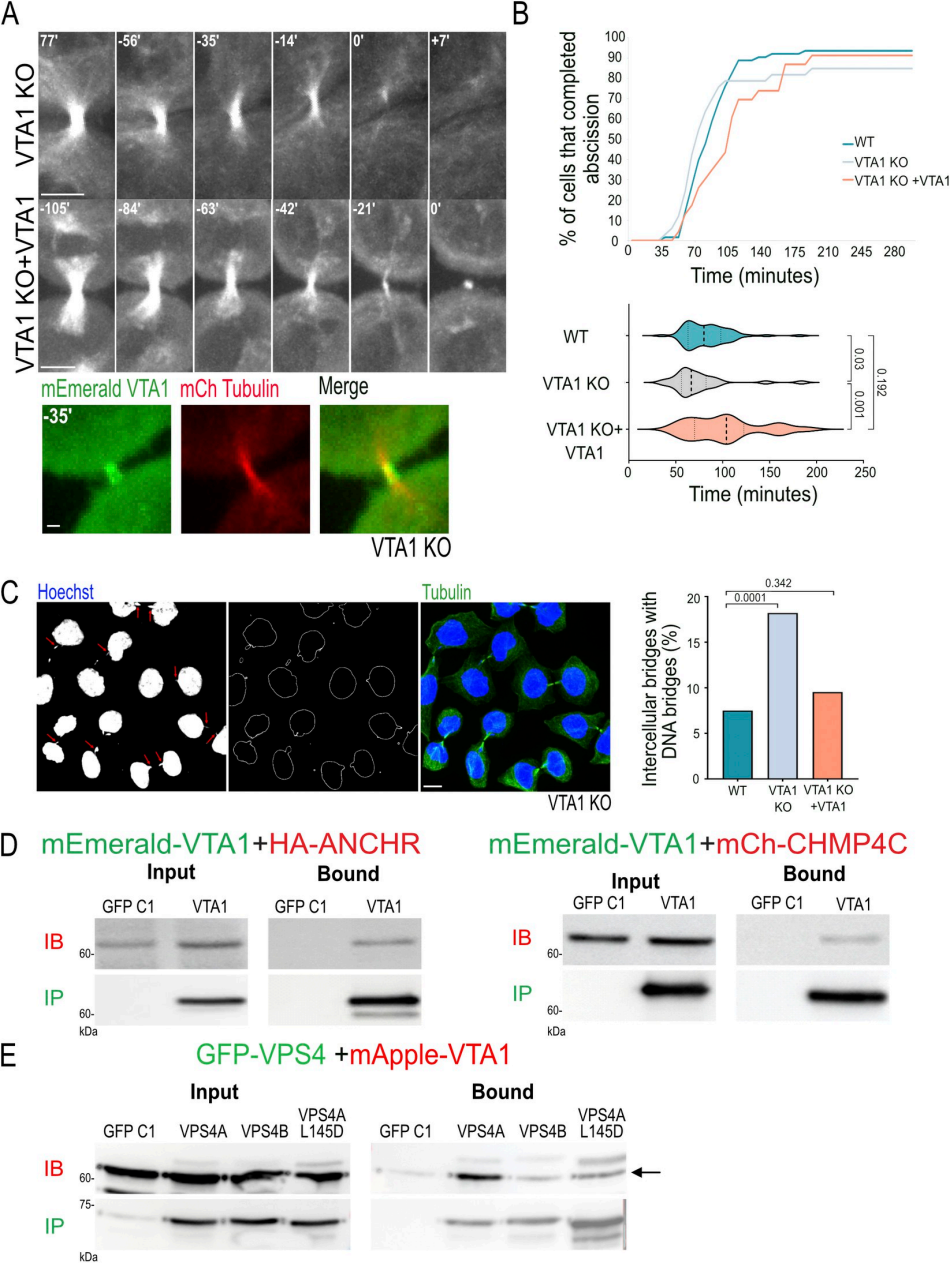
VTA1 is part of the abscission checkpoint complex. (**A**) Abscission is faster in cells depleted of VTA1. Live cell imaging of VTA1 KO cells transfected with GFP-tubulin alone or with mCherry-tubulin and mEmerald-VTA1. Shown are maximum-intensity projections of tubulin labeling from time-lapse movies of representative cells. Time 0 = abscission time. Colored images on bottom panel demonstrate the arrival of VTA1 to the intercellular bridge during abscission in VTA1 KO cells (see also S3 Movie). Scale, 5 μm. (**B**) Duration of abscission (from cleavage furrow formation to microtubule bridge cleavage) as measured for VTA1 KO and VTA1 KO cells expressing exogenous VTA1 are shown on right panels. Top panel, a cumulative plot; bottom panel, a violin plot showing the distribution of abscission times. Averaged duration times of abscission were: VTA1 KO 73.3 ± 30 min, *n* = 32; VTA1 KO + VTA1 102 ± 39 min, *n* = 23 (S1 Data). WT data (reproduced from Fig 1A and 1B) is shown for reference. Data were obtained from at least 3 independent experiments, and *p* values were calculated using Anova (Kruskal–Wallis test). (**C**) Accumulation of chromatin bridges in VTA1 KO cells. Representative images of VTA1 KO cells stained with anti-α-tubulin (green) and Hoechst (blue) are shown. Left panel: DNA staining. Arrows indicate chromatin bridges. Second panel: a Laplacian 2D filter analysis applied to the DNA staining images for better visualization of DNA bridges. Third panel: A merged image of tubulin and DNA staining. Scale, 10 μm. Right panel: A plot showing the percentages of intercellular bridges cells with DNA bridges in WT, VTA1 KO, and VTA1 KO cells expressing mEmerald-VTA1 (WT; *n* = 654, VTA1 KO; *n* = 454, VTA1 KO + VTA1; *n* = 784 cells) (S1 Data). Data was reproduced in at least 2 independent experiments, and *p* values were calculated using chi square. (**D**) VTA1 binds the abscission checkpoint proteins CHMP4C and ANCHR. WT cells co-transfected with mEmerald-VTA1 and HA-ANCHR (left panel) or mCherry-CHMP4C (right panel) were subjected to IP using anti-GFP beads. Immunoblotting: IB fractions anti-HA (left panels) or anti-mCherry (right panels), IP fractions anti-GFP antibodies. (**E**) WT cells co-transfected with the indicated GFP-tagged VPS4 constructs, and mApple-VTA1 were subjected to IP using anti-GFP beads. Immunoblotting: IB fractions anti-VTA1 (arrow indicates the VTA1 band), IP fractions anti-GFP. Note that VTA1 preferentially binds to VPS4A in cells. Data in C and D were reproduced in at least 2 independent experiments. Complete blots of panels D and E are provided in S1 Raw Images. IP, immunoprecipitation.

VTA1 is a co-factor of VPS4, which was shown to stabilize its active, hexameric form [11,12,27–29]. To test whether the regulatory role of VPS4 can be induced by destabilizing the VPS4 hexamer, we examined the effect of VTA1 depletion on VPS4 function in dividing cells using a VTA1 KO cell line [14] (S4A Fig). In native polyacrylamide gel electrophoresis, lower molecular weight bands were obtained for VPS4 in VTA1 KO cells compared to WT cells, supporting decreased levels of hexameric VPS4 in VTA1 KO cells (S4B Fig). Unexpectedly, loss of VTA1 did not result in a delay in abscission but instead led to accelerated abscission (Fig 4A). This phenotype was not associated with a significant increase in multinucleated cells (S1B Fig). Exogenously expressed VTA1 arrived at the intercellular bridge in VTA1 KO cells and reduced the rate of abscission (Fig 4A and 4B). The cellular levels of VPS4A and VPS4B were not significantly affected in these cells and both proteins properly localized at the intercellular bridge, indicating that VTA1 is dispensable for targeting VPS4 to the intercellular bridge (S4C and S4D Fig). Additionally, no changes in the organization and levels of IST1 at the intercellular bridge were observed, suggesting that the ESCRT-III complex is not significantly affected (S4E and S4F Fig). Therefore, despite the canonical role of VTA1 in stabilizing the active VPS4 hexamer, its depletion did not significantly affect the arrival and organization of ESCRT-III-VPS4 proteins to the intercellular bridge or lead to delayed abscission.

**Fig 5:**
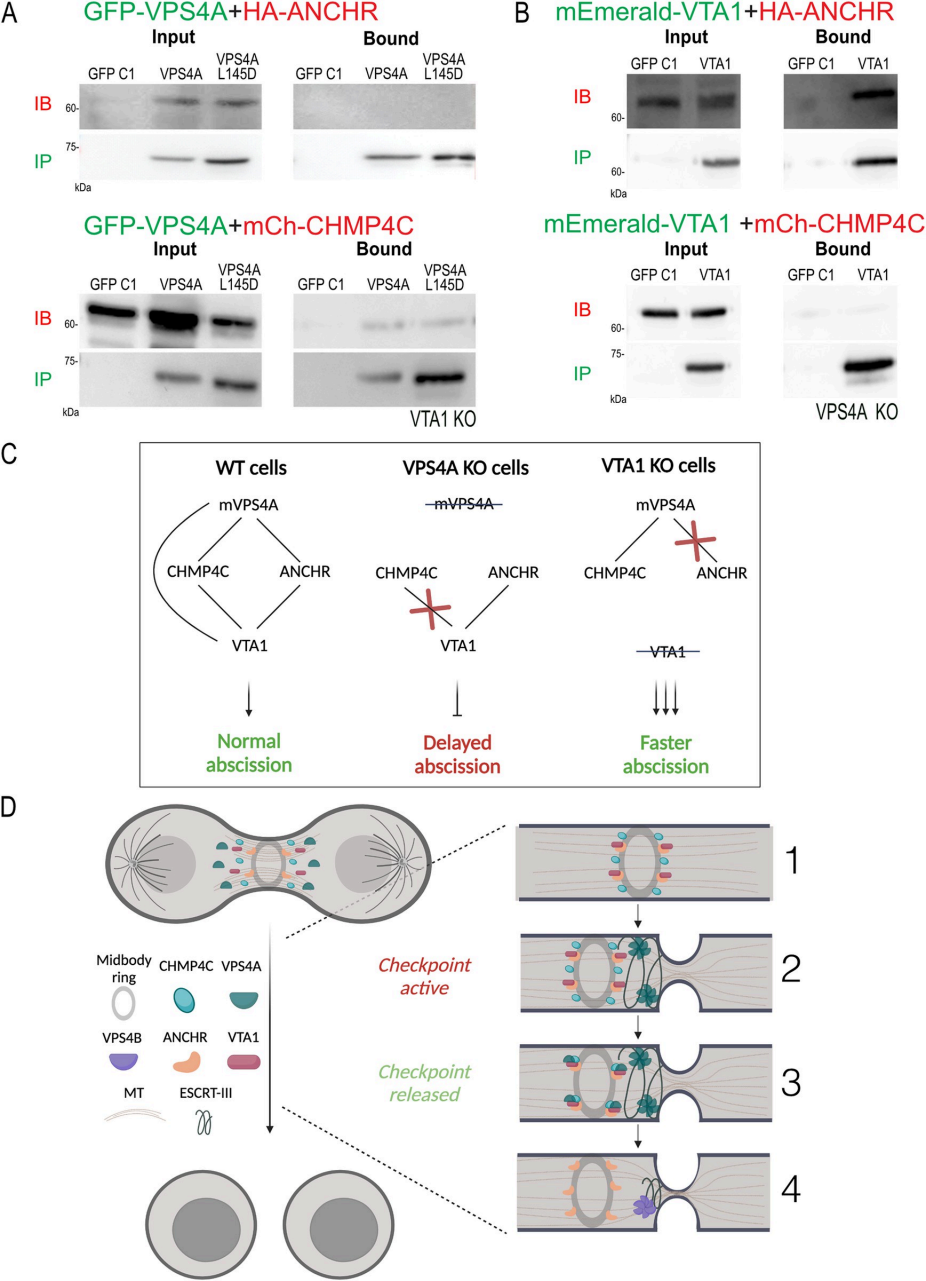
The VPS4A-VTA1 interplay at the abscission checkpoint. (**A, B**) VTA1 KO cells (**A**) or VPS4A KO cells (**B**) co-transfected with the indicated plasmids were subjected to IP using anti-GFP beads. Immunoblotting: IB fractions anti-HA (top panels) or anti-mCherry (bottom panels), IP fractions anti-GFP. Note that VPS4A-ANCHR interactions are abolished in VTA1 KO cells and that VTA1-CHMP4C interactions are abolished in VPS4A KO cells. Data in A and B was reproduced in at least 2 independent experiments. Complete blots are provided in S1 Raw Images. (**C**) A summary of the interactions between VPS4A and VTA1 and the resulting abscission phenotypes in each of cell lines studied. Crossed out text indicate the protein that is depleted the KO cell line; red cross specifies abolished interaction; lines connecting between proteins indicate positive interaction obtained by IP. (**D**) A suggested model for the interplay between VPS4A, VTA1, CHMP4C, and ANCHR at the abscission checkpoint in WT cells. (1) CHMP4C, ANCHR and VTA1 assemble on the midbody ring with ANCHR and VTA1 forming a complex. (2) VPS4A arrives at the intercellular bridge and assembles into hexamers on the membrane, leading to initial bridge constriction. (3) VPS4A continues accumulating at the bridge, resulting in excess of monomeric VPS4A, which binds to CHMPC at the midbody ring. The latter facilitates the formation of an ANCHR-VTA1-VPS4A-CHMP4C complex at the midbody ring. (5) As a result, the abscission checkpoint is released and ESCRT-driven abscission (mediated by VPS4B) progresses, leading to the completion of abscission and the formation of 2 independent daughter cells. ESCRT, endosomal sorting complex required for transport; IP, immunoprecipitation.

Accelerated abscission was previously reported upon depletion of abscission checkpoint proteins, suggesting the involvement of VTA1 in the abscission checkpoint [1,24]. An increase in chromatin bridges, which results from chromosome misseggregation, was previously associated with failure of the abscission checkpoint and specifically with disfunction of the CHMP4C and ANCHR [20,24]. Consistent with a role for VTA1 in the abscission checkpoint, the percentage of intercellular bridges that contained lagging chromatin was significantly increased in VTA1 KO cells, and this phenotype was restored upon exogenous VTA1 expression (Fig 4C). Levels of the abscission checkpoint protein AuroraB were similar in WT and VTA1 KO cells, suggesting that VTA1 does not affect the arrival of AuroraB to the intercellular bridge (S4G Fig). In IP experiments performed in WT cells, VTA1 was found to interact with the checkpoint proteins ANCHR and CHMP4C (Fig 4D). VTA1 could also bind VPS4B and VPS4A, with stronger binding affinity observed for the latter, and was associated with the mVPS4A (Fig 4E). To further investigate the involvement of VTA1 in the VPS4-CHMP4C-ANCHR abscission checkpoint complex, we analyzed the interactions between VPS4, VTA1, ANCHR, and CHMP4C in the KO cell lines (Fig 5A–5C). Interestingly, we found that the interaction between VPS4A and ANCHR was lost in VTA1 KO cells, while its binding to CHMP4C was unperturbed (Fig 5A and 5C). On the other hand, in VPS4A KO cells, VTA1 lost its ability to interact with CHMP4C but maintained binding to ANCHR (Fig 5B and 5C). Collectively, these findings strongly suggest that VTA1 is an integral part of a CHMP4C/ANCHR/VPS4A abscission checkpoint complex and is involved in the regulation of abscission timing.

## Discussion

In this work, we present the first evidence of a distinct role for the 2 VPS4 isoforms in abscission. Our findings support a model in which VPS4B primarily serves the canonical VPS4 function that involves removal of the ESCRT-III monomers from the filament at late abscission stages. The VPS4A isoform hold a dual function: a canonical function involving ESCRT-III monomer exchange at early abscission stages, and a regulatory function, which can be mediated by its monomeric form. We further show that the VPS4A co-factor, VTA1, partner with VPS4A at the abscission checkpoint via binding to checkpoint proteins and facilitating VPS4A-ANCHR interactions. Based on our findings, we propose that VPS4A, which is present in cells at lower levels, acts as a sensor for monitoring the recruitment of VPS4 to the intercellular bridge. When the levels of VPS4A at the bridge reach a certain threshold, the abscission checkpoint is released, resulting in the completion of abscission.

Collectively, our data support a scenario wherein in WT cells VPS4A and VTA1 cooperate with abscission checkpoint proteins to regulate the timing of abscission (Fig 5D). Initially, checkpoint proteins CHMP4C, ANCHR, and VTA1 assemble on the midbody ring, with VTA1 and ANCHR forming a complex. VPS4A arrives at the intercellular bridge, assemble into hexamers on the bridge membrane, and drives the initial constriction of the bridge via remodeling the ESCRT-III polymer. As more VPS4A molecules arrive, monomeric VPS4A accumulates at the intercellular bridge and binds to CHMP4C proteins located at the midbody ring. This accumulation drives the formation of an ANCHR-VTA1-VPS4A-CHMP4C complex at the midbody ring, which facilitates checkpoint release, presumably by promoting the dephosphorylation of CHMP4C [24]. Release of the checkpoint allows for the progression and completion of ESCRT-mediated intercellular bridge abscission, forming 2 fully separated daughter cells. While the precise function of VPS4A and VTA1 in the abscission checkpoint is yet to be fully resolved, the interplay between these proteins at the abscission checkpoint highlights the tight regulation of abscission in cells. It will be interesting to examine, in future studies, whether other VPS4-related cellular processes employs a similar mode of regulation.

Biochemical and structural studies showed that VTA1 stabilizes the VPS4 hexamer and increases ATP hydrolysis by the enzyme [12,29]. Our data show that in the absence of VTA1, the arrival of both VPS4 isoforms and ESCRT-III proteins to the intercellular bridge is unperturbed and that abscission is not inhibited. These data suggest that VTA1’s function in stabilizing the VPS4 hexamer is expansible for the function of VPS4 in abscission. Unexpectedly, our results highlight a role for VTA1 in the abscission checkpoint. VTA1 was found to bind the checkpoint proteins ANCHR and CHMP4C and to mediate the binding of VPS4A to ANCHR. Furthermore, as observed upon depletion of other abscission checkpoint proteins, loss of VTA1 led to faster abscission and to the accumulation of intercellular bridges containing chromatin bridges [1,24]. The findings that VTA1 may be expansible for the canonical function of VPS4 in abscission, but is essential for the regulation of abscission calls for reinvestigation of the function of VTA1 in cells.

The role of VPS4 in the ESCRT pathway has been studied both in vitro and in cells. Perturbation of VPS4 function inhibited ESCRT-mediated processes in cells, including viral budding and cytokinetic abscission [3,14–16,21,22,30,31]. In vitro, VPS4 was shown to induce the disassembly of ESCRT-III filaments and to drive ESCRT-mediated membrane fission in artificial membrane vesicles, indicating that it is executing the final membrane fission event by depolymerization ESCRT-III filaments [16,23,32–36]. VPS4 also facilitated the exchange of ESCRT-III components in the ESCRT filament, both in vitro and in cells, suggesting an earlier function for VPS4 in shaping the ESCRT-III filament [16,23]. Additionally, a role for VPS4 in initiating ESCRT-III assembly was suggested in the context of viral budding and MVB biogenesis [37,38]. Finally, VPS4 was found to be involved in the AuroraB abscission checkpoint, which controls the onset of ESCRT-mediated cytokinetic abscission [20], and to be involved in centrosome maintenance [4,5]. How a single protein can drive such diverse functions was unclear. Our STORM analysis suggests temporally separated functions for VPS4A and VPS4B in abscission. While VPS4A is mainly involved in early abscission stages, presumably in monomeric exchange of the ESCRT-III filament, VPS4B appears to have a role later in the process, when ESCRT-III depolymerization occurs. Such functional discrimination between the 2 VPS4 paralogs provides a potential explanation for the many functions described for VPS4 in cells and raises the possibility that specific VPS4 functions are subjected to different cellular regulations, as demonstrated here for VPS4A. Differences in VPS4A and VPS4B function in cells have been previously reported in the context of viral budding. In particular, it was shown that the HIV virus mainly utilizes the VPS4B isoform to bud out of cells [14,15]. Our observations that the cellular levels of VPS4B are significantly higher than VPS4A and that VPS4A is involved in the regulation of abscission provide a logical explanation for these observations. Utilizing the VPS4B isoform is advantageous for the virus, as this isoform is more abundant in cells and less involved in cellular regulation.

VPS4 has been previously associated with various pathologies, including cancer and neurodevelopmental disorders. However, while the deletion of VPS4B has been linked to cancer mostly indirectly due to its location on chromosome 18 [8], specific heterozygous point mutations in VPS4A were sufficient to induce severe pathologies [6,7]. Two independent studies have demonstrated that individuals carrying single mutations in the VPS4A gene developed dyserythropoietic anemia and severe neurodevelopmental delays [6,7]. These conditions were associated with structural brain abnormalities, intellectual disability (ID), deafness, cataracts, and visual dysfunction. In one of the studies, fibroblasts taken from patients showed defects in centrosome number, primary cilia morphology, and cell cycle progression [6]. Interestingly, EGFR degradation, which relies on ESCRTs for internalization to multivesicular bodies, remained normal, suggesting that specific ESCRT-dependent cellular processes are more sensitive to the loss of VPS4A. Notably, enlarged endosomes with abnormal IST1 accumulations, but not other ESCRT-III proteins, were observed in these cells, strongly indicating a link between VPS4A and IST1. These findings are consistent with our STORM data, which demonstrated that IST1 accumulates at the intercellular bridge upon VPS4A loss. In the second study, blood cells from patients displayed bi-nucleated cells and accumulation of cytokinetic bridges [7]. iPSC cells derived from these patients exhibited late cytokinesis defects accompanied by mislocalization of VPS4 at the intercellular bridge. The crucial role of VPS4A in neurodevelopment suggests that its cellular regulation is paramount for proper neuronal development and function. Despite the presence of a normal VPS4B gene and adequate VPS4B levels in these patients, the disruption of VPS4A seems to impact disease progression significantly, indicating the uniqueness of VPS4A’s functions in these pathologies. Yet, so far, a mechanistic explanation for these observations has been lacking. Our data show that depletion of VPS4A lead to a severe abscission delay, albeit high endogenous VPS4B cellular levels, and that while exogenous expression of VPS4A can fully rescue the abscission delay over expression of VPS4B cannot. By performing an in-depth investigation of the role of each VPS4 isoform in abscission our findings provide, for the first time, a plausible mechanistic explanation for the observed pathologies. We propose that patients who carry normal VPS4B and mutated VPS4A genes develop pathologies due to the disruption of VPS4A-mediated cellular regulation, which is essential for neurodevelopment and cannot be compensated by VPSB. This VPS4A-mediated regulation may be directly related to the abscission checkpoint regulation, reported here, or may involve other regulatory roles of VPS4A in cells that are yet to be characterized. Further investigation of the regulatory functions of VPS4A in cells could open new avenues for targeted therapeutic interventions for these severe pathologies.

## Methods

### Cell culture and transfection

HeLa cells were grown in DMEM supplemented DMEM with 10% fetal bovine serum (FBS), 2 mM glutamine, 10,000 U/mL penicillin, 10 mg/mL streptomycin at 37°C, and 5% CO2. Transfection was carried out by using Lipofectamine 2000 (Life Technologies), PolyJet (SignaGen Laboratories), or JetPrime transfection kit (Polyplus), according to manufacturer’s guidelines.

### Plasmid constructs

Fluorescently tagged versions of human VPS4A and VPS4B were previously cloned in our laboratory as described in Ott and colleagues [5]. A monomeric VPS4A version (L145D) was designed based on the previously published monomeric yeast VPS4 (VPS4 L151D) (S3A Fig) [26]. The point mutation at position L145 was introduced by overlapping PCR. GFP-ANCHR: Full-length human ANCHR conjugated to GFP was a kind gift by Harald Stenmark (Centre for Cancer Biomedicine, Faculty of Medicine, Oslo University Hospital, Norway) [20]. An HA tagged version (HA-ANCHR) was generated by subcloning ANCHR sequence into a 3xHA-C1 plasmid. mEmerald/ mApple -VTA1: Full-length human VTA1 was amplified by PCR and cloned to mEmerald or mApple -C1 vectors (Clontech). mCh-CHMP4C, GFP–α-tubulin, and mCherry–α-tubulin were generated as previously described [18]. All constructs were confirmed by sequencing.

### Genome editing

For CRISPR/Cas9-mediated gene disruption, guide RNAs (gRNAs) Guide RNAs (gRNAs) targeting VPS4A, VPS4B, and VTA1 were subcloned into the lentiCRISPR plasmid (Addgene, #49535). Following transfection and puromycin selection, single clones were isolated and expanded. To confirm the efficacy of protein knockouts, western blot analysis was performed. The gRNA sequences employed were as follows: CACCGAGTGCGTGCAGTACCTAGAC for targeting VPS4A, CACCGCAAACAGAAAGCGATAGATC for targeting VPS4B, and CACCGGCATGACAAGCGAGACCCTG for targeting VTA1.

KO cells were sequenced, and mutations were identified (S3 Data).

Protein depletion was confirmed using western blot analysis (S1A and S4A Figs).

### Cell synchronization

Double thymidine block was performed as previously described [21]. In short, HeLa cells were plated at 10% confluency on an Ibidi Glass Bottom Dish 35 mm (Martinsried, Germany) and treated with 2 mM thymidine (Sigma, T1895-1G) for 18 h to induce the first block. After incubation, cells were washed once with PBS and grown in fresh media for 9 h. Cells were then supplemented for an additional 15 h with 2 mM thymidine, washed once with PBS, and grown in fresh media for 10.5 to 12.5 h to enrich the percentages of intercellular bridges.

### Immunoprecipitation (IP)

IP was carried out using the GFP-Trap Agarose KIT (ChromoTek), following the manufacturer’s instructions with minor modifications. Transfected HeLa cells were harvested 24 h after transfection and washed with PBS. The cells were then lysed on ice for 30 min using a lysis buffer containing 10 mM Tris/Cl (pH 7.5), 100 mM NaCl, 0.5 mM EDTA, 0.5% NP40, 1 mM PMSF, and a complete protease inhibitor cocktail (Roche Diagnostics). Cell lysates were then centrifuged at 20,000 g for 10 min at 4°C, and supernatants were diluted in a buffer consisting of 10 mM Tris/Cl (pH 7.5), 150 mM NaCl, 0.5 mM EDTA, 1 mM PMSF, and a complete protease inhibitor cocktail. The diluted lysates were incubated with GFP-Trap-A beads for 1 h. Subsequently, the lysates and beads were centrifuged at 2,500 g for 2 min at 4°C to separate the unbound fraction from the bound fraction. Samples were heated at 95°C for 5 min in Laemmli sample buffer and resolved by SDS-PAGE followed by western blot analysis.

### Western blot

HeLa cells were lysed as described above or in RIPA lysis buffer [150 mM NaCl, 1% NP-40, 0.5% deoxycholate, 0.1% SDS, 50 mM Tris (pH 8.0)] supplemented with complete protease inhibitor (Roche Diagnostics) for 30 min at 4°C. Total protein concentrations of the lysates were determined using BCA Protein Assay Kit (Pierce Biotechnology). Samples were heated at 95°C for 5 min in Laemmli sample buffer and resolved by SDS-PAGE. Membranes were stained with primary antibodies for 16 h at 4°C using the following antibodies: anti-GFP (Applied Biological Materials, G096, 1:1,000), anti mCherry (Novus Biologicals, NBP2-25157, 1:1,000-WB), anti VTA1 (Thermo Fisher, PA5-21831 1:1,500), anti-HA (Applied Biological Materials, G166, 1:4,000), anti-VPS4 (Sigma-Aldrich, SAB4200025, 1:500). Then, a secondary anti-rabbit or anti-mouse-peroxidase antibodies (1:10,000; Jackson ImmunoResearch) were applied for 1 h.

### Native PAGE

HeLa wild-type (WT) and VTA1 knockout (KO) cells were lysed using a hypotonic buffer consisting of 10 mM HEPES (pH 7.9), 1.5 mM MgCl, and 10 mM KCl. A total of 30 μl cell extracts were loaded on native polyacryamide gel. The membrane was then incubated with primary antibodies overnight at 4°C, followed by incubation with anti-rabbit peroxidase-conjugated secondary antibodies for 1 h at 1:10,000 dilution (Jackson ImmunoResearch).

### Immunofluorescence

HeLa cells were washed with PBS, fixed with 4% paraformaldehyde (PFA), permeabilized with 0.5% Triton X-100 for 10 min, and blocked with 10% FBS for 15 min. Fixed cells were stained with the primary antibodies: anti-CHMP4B (Proteintech, 13683-1-AP, 1:50), anti-IST1 (Proteintech, 51002-1-AP, 1:50), anti-tubulin (Sigma, T6199 1:1,000), anti-AurB (abcam, ab2254, 1:1,000), and anti-AurBpT232 (Rockland, 600-401-667S, 1:500-IF), as indicated, and subjected to fluorescently-tag secondary antibodies staining (Alexa Fluor 488 or Alexa Fluor 594, Life Technologies). Finally, cells were mounted with Fluoromount-G (SouthernBiotech, Birmingham, Alabama, United States of America) and imaged.

### Live cell imaging

HeLa cells were plated at low density in four-well chamber slides (Ibidi, Martinsried, Germany), transfected 24 h later with the indicated plasmids, and imaged 24 to 48 h later. Z-stacks of selected low-expressing cells undergoing cytokinesis were collected at the specified intervals using a fully incubated confocal spinning-disk microscope (Marianas; Intelligent Imaging, Denver, Colorado, USA) with a 63× oil objective (numerical aperture, 1.4) and were video recorded on an electron-multiplying charge-coupled device camera (Evolve; pixel size, 0.079 μm; Evolve; Photometrics). Image processing and analysis were done using SlideBook version 6 (3I Inc.). Duration of abscission was quantified by measuring the time interval from furrow ingression to complete separation of the 2 daughter cells, as previously described [22,39]. Care was taken to include in the analysis only cells in which these time points could be clearly specified and the entire process could be followed.

### Structured illumination microscopy (SIM)

Image acquisition and processing was performed as previously described [40]. In short, cells were plated at low density on high-resolution #1.5 coverslips (Marienfeld, Lauda-Konigshfen, Germany) and fixed using 4% PFA. Cells were further subjected to immunostaining as described above. 3D SIM imaging of low expressing cells was performed using the ELYRA PS.1 microscope (Carl Zeiss MicroImaging). Thin z-sections (0.11 to 0.15 μm) of high-resolution images were collected in 3 rotations and 5 phases for each channel. Image reconstruction and processing were performed in ZEN (Carl Zeiss MicroImaging).

### STORM experiments

Synchronized cells were washed with PBS, fixed with 3% PFA and 0.1% glutaraldehyde, and permeabilized and blocked with 0.2% Triton X-100 with 3% BSA. Cells were then stained with tubulin and IST1 antibodies. Anti-mouse Alexa Fluor 488 and anti-rabbit Alexa Fluor 647 (Life Technologies, 1:1,500) were used as secondary antibodies, respectively. Cells were subjected to a second fixation (as above). Acquisition was performed in STORM buffer containing 7 μm glucose oxidase (Sigma), 56 nM catalase (Sigma), 5 mM cysteamine (Sigma), 50 mM Tris, 10 mM NaCl, and 10% glucose (pH 8) using a Zeiss Elyra ELYRA PS.1 microscope in wide-field mode (100× N.A 1.46 oil immersion objective). Excitation was induced using a 647 nm laser (5 kW/cm^2^). A total of 10,000 frames were acquired for each dataset with an acquisition time of 35 ms per frame. Experiment was repeated 5 times. To avoid variations resulting from the STORM buffer, all conditions were tested in each of the 5 experimental repeats (early/late bridges; WT, VPS4A KO, and VPS4B KO cells). Analysis was done using ZEN 2011 software (Zeiss, Germany). Datasets were filtered similarly for all conditions according to the following parameters: peak mask size – 5, peak intensity to noise – 7, drift correction – 3, localization precision – 1-30 nm, minimal number of photons – 500, first frame – 1,000 and up. Quantification of IST1 density at the intercellular bridge was performed as follows: An ROI surrounding IST1 localization at each side of the intercellular bridge was depicted manually (see example in Fig 2B, WT image). For each ROI, the number of localizations was determined. To avoid bias resulting from the buffer conditions number of localizations were normalized for each experimental repeat by dividing the value obtained for each ROI by the maximal value obtained in the specific experimental repeat (across all conditions tested). Localization densities at the intercellular bridge were calculated for each ROI by dividing the normalized number of localizations by the area of the ROI. Plots of the ROI area and normalized number of locations obtained for each condition are available in S2B Fig.

### Statistical analysis

Statistical analysis was performed using GraphPad Prism version 9.00 for Windows (La Jolla, California, USA). Normality was tested using the D’Agostino-Pearson test. Statistical analysis was performed using either Student *t* test (two-group comparison), Chi-square test, or Kruskal–Wallis test followed by Dunn’s multiple comparisons for multi-sample comparison, as indicated in the figure legends. Differences with *P* values less than 0.05 were considered significant. Bars in all plots indicate standard deviation of the dataset.

## Supporting information

S1 Fig (**A**) Levels of VPS4A and VPS4B in knockout cells. Western blot analysis was performed on HeLa cells depleted of either VPS4A or VPS4B. The depletion was achieved by transfecting the cells with guide RNA (gRNA) targeting the specific sequence of VPS4A or VPS4B, along with Cas9 and a selection marker. After puromycin selection, the cells were isolated, harvested, and 30 μg of total protein was loaded on each gel lane. Protein expression levels were then detected using anti-VPS4 antibodies. Total protein loading was verified using in-gel 2,2,2-trichloroethanol (TCE) fluorescence, by supplementing 1% TCE staining to the SDS-PAGE gel. (B) WT VPS4A KO and VTA1 KO cells were plated on coverslips, stained with anti-tubulin and Hoechst and the level of multinucleated cells (left panel) and chromatin bridges (right panel) was calculated for each condition. WT *n* = 654 cells, VPS4A KO *n* = 634 cells, VTA1 KO = 454 cells (S1 Data), and *p* values were calculated using chi square. (**C**) WT cells transfected with either tubulin-GFP or tubulin-mCherry were subjected to live cell imaging. Z slices of dividing cells were captured at 7-min intervals using a confocal spinning-disk microscope. Duration of abscission (from cleavage furrow formation to microtubule bridge cleavage) was measured for each cell and plotted in a cumulative plot. Note that abscission time was not affected by the fluorescent protein conjugated to tubulin. Data for each condition were obtained from at least 2 independent experiments; *n* = 13 cells (S1 Data). (**D**) VPS4A (top panel) and VPS4B (bottom panel) localize to the intracellular bridge in WT cells. Shown are intercellular bridges of cells that were transfected with GFP-VPS4 (A or B, as indicated, green), fixed 24 h later, and stained with anti-tubulin antibodies (red). Left panels: zoom-out images (scale, 10 μm). Zoom-in images of the area in white squares on left panels are shown on the right (scale, 10 μm). Shown are maximum-intensity projection images of representative cells. Data was reproduced in at least 3 independent experiments. (**E**) WT and VPS4A KO cells were transfected with either GFP-VPS4A or GFP-VPS4B, as indicated, and subjected to western blot analysis using anti-VPS4 or anti-GFP antibodies. Equal total protein amounts were loaded in each lane. Results using anti-VPS4 show that the level of exogenous expression is considerably higher than endogenous VPS4 levels. Anti-GFP antibody staining show that similar expression levels are obtained for VPS4A and VPS4B upon exogenous expression. (**F**) The endogenous expression levels of VPS4B are significantly higher compared to VPS4A. Purified VPS4A-GFP (left) and VPS4B-GFP (right) were loaded on the gel at gradually increasing known concentrations, as indicated, together with cell extracts of WT HeLa cells (first and second lanes on each gel) and stained using anti-VPS4 antibodies which recognizes both VPS4 isoforms. Band intensities were measured for purified VPS4A and VPS4B, and calibration curves were constructed (bottom panels) and used to determine the endogenous expression levels of VPS4A and VPS4B in WT HeLa cells by measuring the band intensities of the protein extracts (S1 Data). This analysis resulted in a ~ 5:1 access of VPS4B over VPS4A in HeLa cells.
(PNG)

S2 Fig (**A**) SIM imaging shows normal ESCRT-III localization at the intercellular bridge in VPS4 KO cells. WT (top panel), VPS4A KO (middle panel), and VPS4B KO (bottom panel) cells were stained with anti-α-tubulin (green) and anti-CHMP4B (red) antibodies and imaged by SIM. Maximum projection images of early (left) and late (right) intercellular bridges are shown. Scale, 1 μm. (**B**) STORM measurements obtained for IST1 at early and late intercellular bridges of WT and KO cells. An ROI containing the IST1 signal at either side of the intercellular bridge was manually selected and measured (see ROI in Fig 2B). Top panel: number of localizations measured at ROI. Data was normalized for each experiment to avoid bias resulting from buffer conditions (see Methods section). Bottom panel: area calculated for each ROI at the different conditions. Area and localization values measured for each ROI were used for density calculations presented in Fig 2B. Raw data are provided in S2 Data.
(TIFF)

S3 Fig (**A**) Sequence alignment of human VPS4A and yeast VPS4. Rectangle indicates the residue the conserved L residue that was subjected to point mutation. (**B**) A violin plot showing the distribution of abscission times in WT, VPS4A KO, and VPS4A KO expressing mVPS4A mutant. Corresponds to Fig 3D.
(TIFF)

S4 Fig (**A**) Levels of VTA1 in KO cells. Western blot analysis was performed on HeLa and KO cells, as indicated, and 30 μg of total protein was loaded on each gel lane. Protein expression levels were detected using anti-VTA1 antibodies. (**B**) WT and VTA1 KO cells were lysed, and an equal total volume was run on a native polyacrylamide gel. The resulting gel was then transferred to a membrane and probed with an anti-VPS4 antibody. Note that lower molecular weight bands are observed in VTA1 KO cells compared to WT cells. (**C**) The absence of VTA1 does not affect the total levels of VPS4. The indicated cells were harvested and subjected to a lysis buffer. Western blot analysis was performed on HeLa and KO cells, as indicated. Equal protein amounts were loaded in each lane, and the membrane was probed with anti-VPS4 antibodies. (**D**) VPS4A and VPS4B arrive to the intercellular bridge in VTA1 KO cells. Shown are intercellular bridges of cells that were transfected with GFP-VPS4 (A or B as indicated, green), fixed 24 h later, and stained with anti-tubulin antibodies (red). Left panels: zoom-out images. Zoom-in images of the area in white squares on left panels are shown on the right. Shown are maximum-intensity projection images of representative cells. Scale, 10 μm. Data was reproduced in at least 2 independent experiments. (**E**) SIM imaging shows normal ESCRT-III localization at the intercellular bridge in VTA1 KO cells. VTA1 KO cells were stained with anti-α-tubulin (green) and anti-IST1 (red) antibodies and imaged by SIM (scale bar = 1 μm). Maximum projection images of early (top) and late (bottom) intercellular bridges are shown. Data was reproduced in at least 2 independent experiments. Scale, 1 μm. (**F, G**) Fixed VTA1 KO cells subjected to co-immunostaining using anti-tubulin antibodies (green) and either anti-IST1 (F, red), anti-AurB (top panel G, red), or anti-AurBpT232 (bottom panel G, red). Cells were imaged using confocal microscopy, and maximum-intensity projections images are shown. Left panels: zoom-out images (scale, 10 μm). Zoom-in images of the area in white squares on left panels are shown on the right (scale, 5 μm). The total intensities of the indicated proteins at the cell bridge were quantified for each of the tested cell types and shown on the right (IST1: WT; *n* = 30, VTA1 KO; *n* = 36. AurB: WT; early *n* = 15, late *n* = 23, VTA1 KO; early *n* = 22, late *n* = 18. AurBpT232: WT; early *n* = 18, late *n* = 19, VTA1 KO; early = 18, late *n* = 34) (S1 Data). Data was obtained from 2 independent experiments, for each condition. Statistical analysis was performed for IST1 (F) using unpaired two-tailed Mann–Whitney U test and for AurB and AurBpT232 (G) using Dunn’s test.
(TIFF)

S1 Movie VPS4A arrives at the intercellular bridge in VPSS4A KO cells and rescues the abscission delay phenotype. VPS4A KO cells transfected with mCh-tubulin (red) and GFP-VPS4A (green) were imaged in 3D at 7 min intervals. Shown are maximum projection images. Scale, 1 μm.
(MP4)

S2 Movie VPS4B arrives at the intercellular bridge in VPSS4A KO cells and partially rescues the abscission delay phenotype. VPS4A KO cells transfected with mCh-tubulin (red) and GFP-VPS4B (green) were imaged in 3D at 7 min intervals. Shown are maximum projection images. Scale, 1 μm.
(MP4)

S3 Movie VTA1 arrives at the intercellular bridge in VTA1 KO cells and rescues the abscission phenotype. VTA1 KO cells transfected with mCh-tubulin (red) and GFP-VTA1 (green) were imaged in 3D at 7 min intervals. Shown are maximum projection images. Scale, 1 μm.
(MP4)

S1 Data Raw data of all quantitative measurements. (XLSX)

S2 Data Raw data of STORM experiments. (XLSX)

S3 Data Sequencing results of KO cells. (XLSX)

S1 Raw Images Complete blots. (PDF)

## Abbreviations

CDA: congenital dyserythropoietic anemia
ESCRT: endosomal sorting complex required for transport
FBS: fetal bovine serum
IP: immunoprecipitation
MVB: multivesicular body
SIM: structured illumination microscopy
STORM: stochastic optical reconstruction microscopy
