# Supplementary figures and images for: The human AAA-ATPase VPS4A isoform and its co-factor VTA1 have a unique function in regulating mammalian cytokinesis abscission
Source: PLoS Biol. 2024 Apr 30;22(4):e3002327. doi: 10.1371/journal.pbio.3002327 (PMC11086821; doi:10.1371/journal.pbio.3002327)

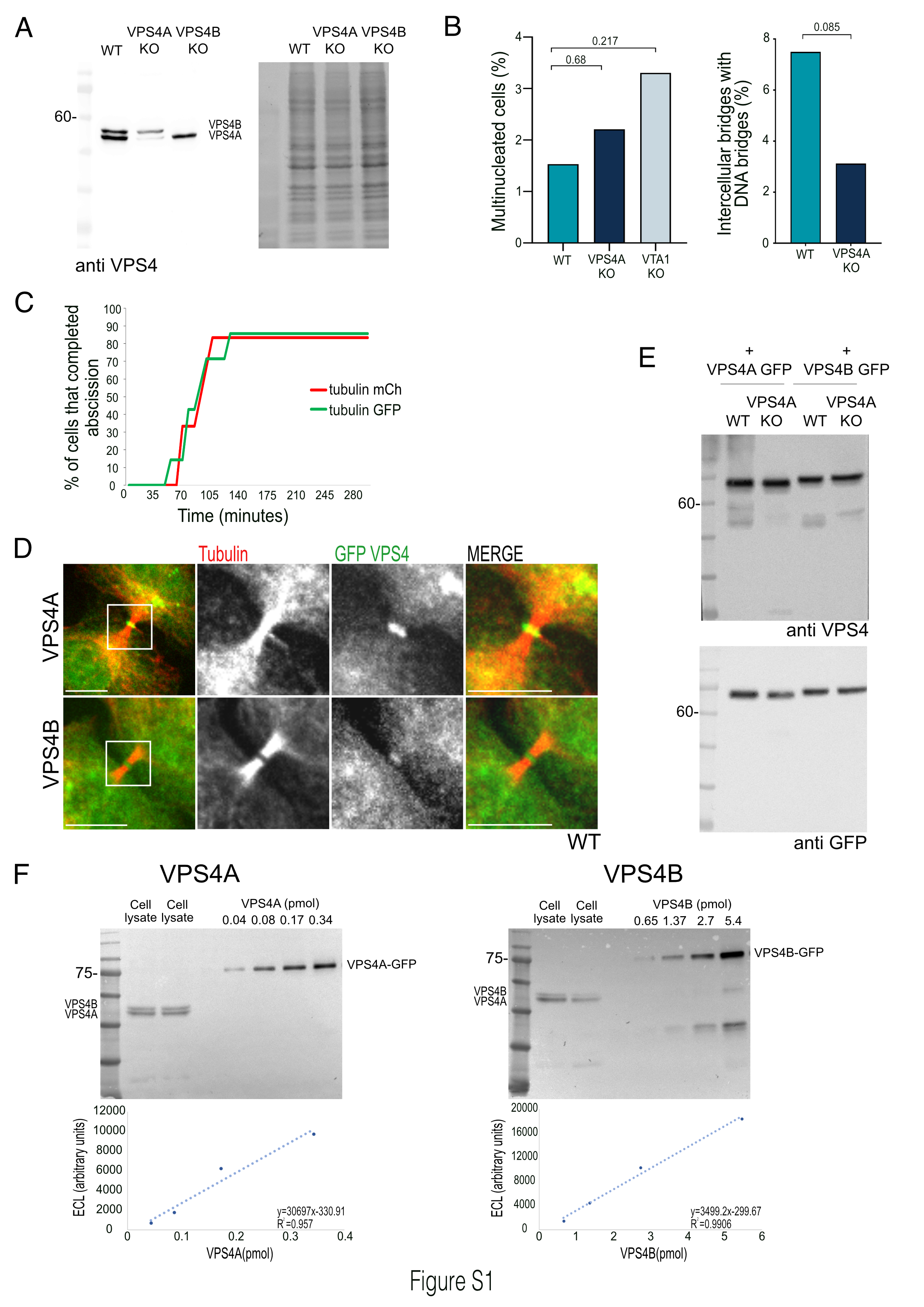

Supplement: S1 Fig — (A) Levels of VPS4A and VPS4B in knockout cells. Western blot analysis was performed on HeLa cells depleted of either VPS4A or VPS4B. The depletion was achieved by transfecting the cells with guide RNA (gRNA) targeting the specific sequence of VPS4A or VPS4B, along with Cas9 and a selection marker. After puromycin selection, the cells were isolated, harvested, and 30 μg of total protein was loaded on each gel lane. Protein expression levels were then detected using anti-VPS4 antibodies. Total protein loading was verified using in-gel 2,2,2-trichloroethanol (TCE) fluorescence, by supplementing 1% TCE staining to the SDS-PAGE gel. (B) WT VPS4A KO and VTA1 KO cells were plated on coverslips, stained with anti-tubulin and Hoechst and the level of multinucleated cells (left panel) and chromatin bridges (right panel) was calculated for each condition. WT n = 654 cells, VPS4A KO n = 634 cells, VTA1 KO = 454 cells (S1 Data), and p values were calculated using chi square. (C) WT cells transfected with either tubulin-GFP or tubulin-mCherry were subjected to live cell imaging. Z slices of dividing cells were captured at 7-min intervals using a confocal spinning-disk microscope. Duration of abscission (from cleavage furrow formation to microtubule bridge cleavage) was measured for each cell and plotted in a cumulative plot. Note that abscission time was not affected by the fluorescent protein conjugated to tubulin. Data for each condition were obtained from at least 2 independent experiments; n = 13 cells (S1 Data). (D) VPS4A (top panel) and VPS4B (bottom panel) localize to the intracellular bridge in WT cells. Shown are intercellular bridges of cells that were transfected with GFP-VPS4 (A or B, as indicated, green), fixed 24 h later, and stained with anti-tubulin antibodies (red). Left panels: zoom-out images (scale, 10 μm). Zoom-in images of the area in white squares on left panels are shown on the right (scale, 10 μm). Shown are maximum-intensity projection images of represe [file pbio.3002327.s001.png]

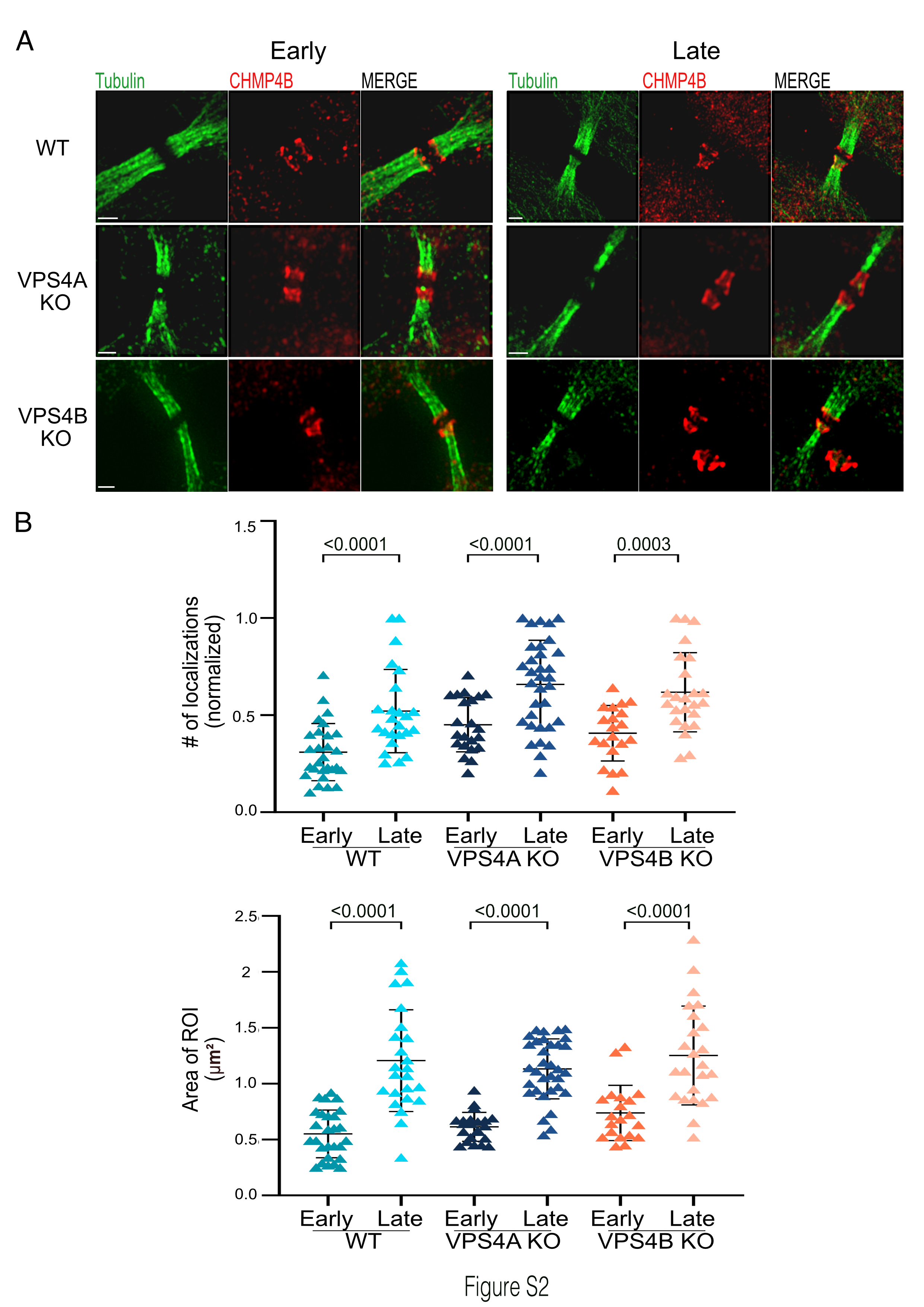

Supplement: S2 Fig — (A) SIM imaging shows normal ESCRT-III localization at the intercellular bridge in VPS4 KO cells. WT (top panel), VPS4A KO (middle panel), and VPS4B KO (bottom panel) cells were stained with anti-α-tubulin (green) and anti-CHMP4B (red) antibodies and imaged by SIM. Maximum projection images of early (left) and late (right) intercellular bridges are shown. Scale, 1 μm. (B) STORM measurements obtained for IST1 at early and late intercellular bridges of WT and KO cells. An ROI containing the IST1 signal at either side of the intercellular bridge was manually selected and measured (see ROI in Fig 2B). Top panel: number of localizations measured at ROI. Data was normalized for each experiment to avoid bias resulting from buffer conditions (see Methods section). Bottom panel: area calculated for each ROI at the different conditions. Area and localization values measured for each ROI were used for density calculations presented in Fig 2B. Raw data are provided in S2 Data. (TIFF) [file pbio.3002327.s002.tiff]

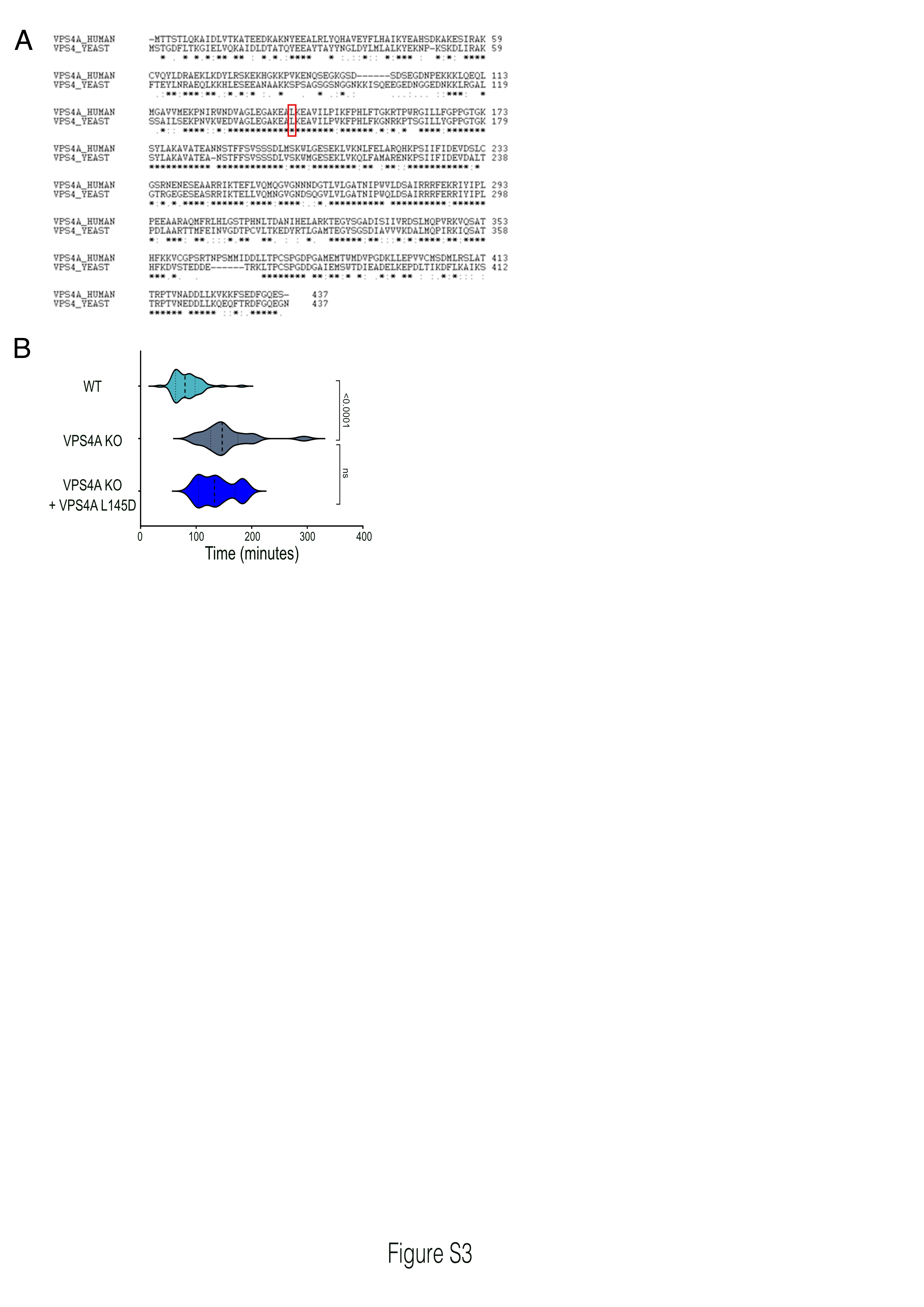

Supplement: S3 Fig — (A) Sequence alignment of human VPS4A and yeast VPS4. Rectangle indicates the residue the conserved L residue that was subjected to point mutation. (B) A violin plot showing the distribution of abscission times in WT, VPS4A KO, and VPS4A KO expressing mVPS4A mutant. Corresponds to Fig 3D. (TIFF) [file pbio.3002327.s003.tiff]

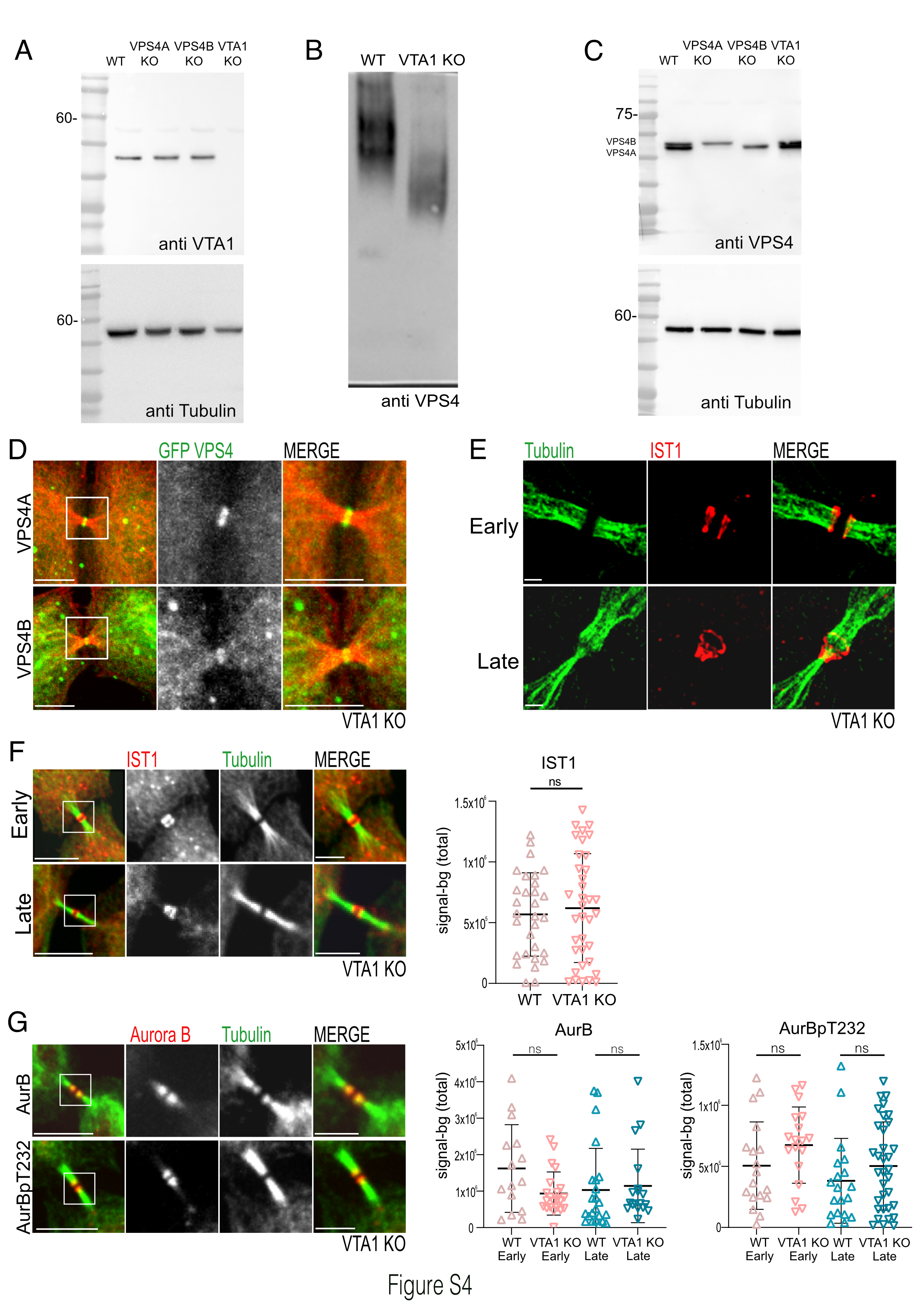

Supplement: S4 Fig — (A) Levels of VTA1 in KO cells. Western blot analysis was performed on HeLa and KO cells, as indicated, and 30 μg of total protein was loaded on each gel lane. Protein expression levels were detected using anti-VTA1 antibodies. (B) WT and VTA1 KO cells were lysed, and an equal total volume was run on a native polyacrylamide gel. The resulting gel was then transferred to a membrane and probed with an anti-VPS4 antibody. Note that lower molecular weight bands are observed in VTA1 KO cells compared to WT cells. (C) The absence of VTA1 does not affect the total levels of VPS4. The indicated cells were harvested and subjected to a lysis buffer. Western blot analysis was performed on HeLa and KO cells, as indicated. Equal protein amounts were loaded in each lane, and the membrane was probed with anti-VPS4 antibodies. (D) VPS4A and VPS4B arrive to the intercellular bridge in VTA1 KO cells. Shown are intercellular bridges of cells that were transfected with GFP-VPS4 (A or B as indicated, green), fixed 24 h later, and stained with anti-tubulin antibodies (red). Left panels: zoom-out images. Zoom-in images of the area in white squares on left panels are shown on the right. Shown are maximum-intensity projection images of representative cells. Scale, 10 μm. Data was reproduced in at least 2 independent experiments. (E) SIM imaging shows normal ESCRT-III localization at the intercellular bridge in VTA1 KO cells. VTA1 KO cells were stained with anti-α-tubulin (green) and anti-IST1 (red) antibodies and imaged by SIM (scale bar = 1 μm). Maximum projection images of early (top) and late (bottom) intercellular bridges are shown. Data was reproduced in at least 2 independent experiments. Scale, 1 μm. (F, G) Fixed VTA1 KO cells subjected to co-immunostaining using anti-tubulin antibodies (green) and either anti-IST1 (F, red), anti-AurB (top panel G, red), or anti-AurBpT232 (bottom panel G, red). Cells were imaged using confocal microscopy, and maximum-intensity projections images are [file pbio.3002327.s004.tiff]
