# Supplementary material for: The human AAA-ATPase VPS4A isoform and its co-factor VTA1 have a unique function in regulating mammalian cytokinesis abscission
Source: PLoS Biol. 2024 Apr 30;22(4):e3002327. doi: 10.1371/journal.pbio.3002327 (PMC11086821; doi:10.1371/journal.pbio.3002327)

Differential role for VPS4 isoforms in cytokinetic abscission confers a regulatory function for VPS4A and VTA1 in mammalian cells

Inbar Dvilansky[2,1$], Yarin Atlaras[1,2$], Nikita Kamenetsky[2,1], Dikla Nachmias[2,1], Natalie Elia[1,2*]

1 Department of Life Sciences, Ben-Gurion University of the Negev, Beer Sheva 84105, Israel
2 National Institute for Biotechnology in the Negev (NIBN), Ben-Gurion University of the Negev, Beer Sheva 84105, Israel

[$] Inbar Dvilansky and Yarin Atlaras contributed equally to this work.

* Corresponding author:
Natalie Elia
Phone: 972-8-6428735
Email: elianat@post.bgu.ac.il

Running title: Role of VPS4 isoforms in cytokinetic abscission

RAW IMAGES FOR BLOTS

Complete blots Fig. 3A

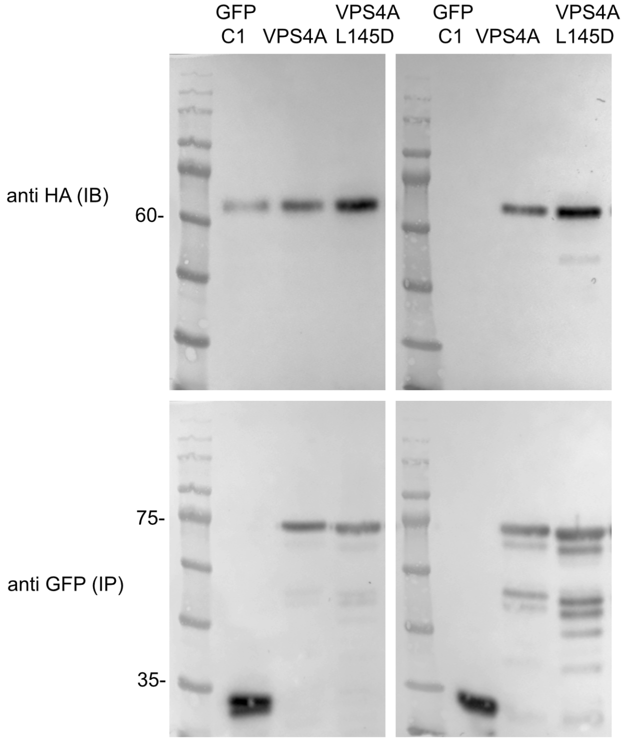

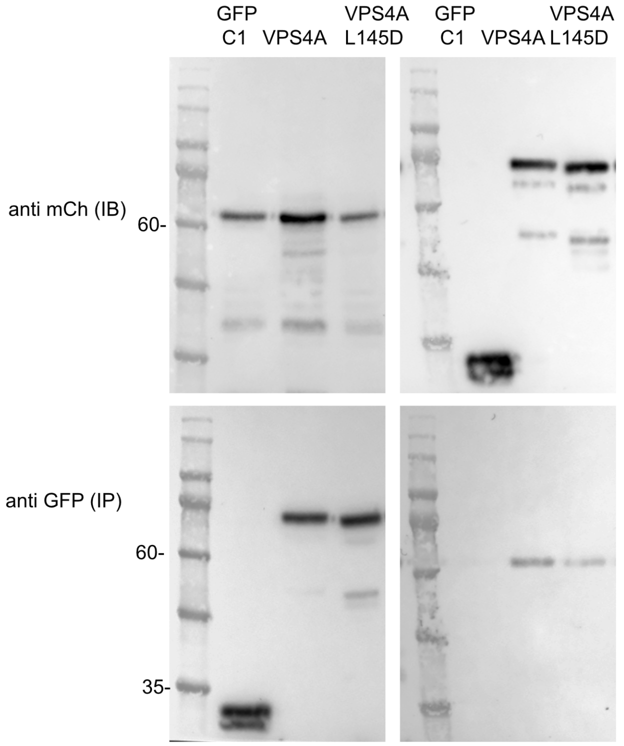

Complete blots Fig. 3B

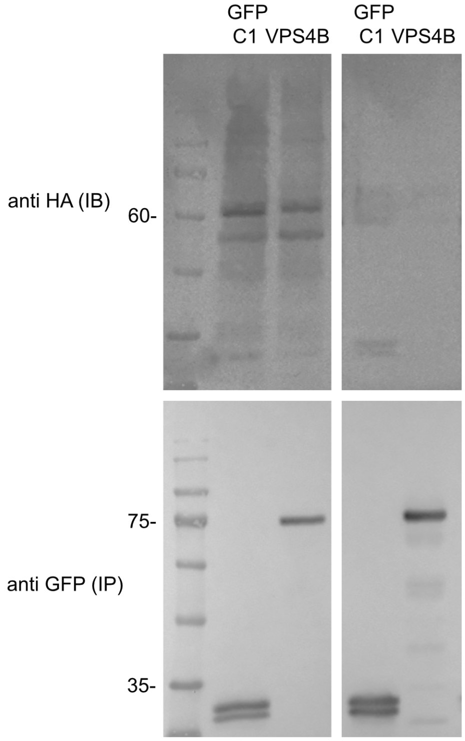

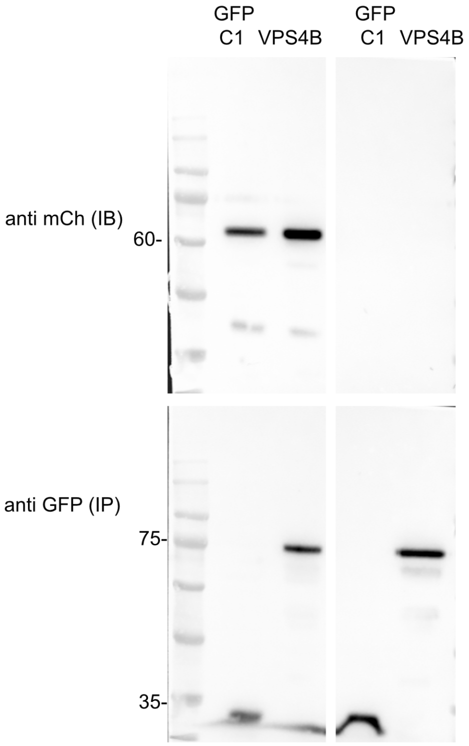

Complete blots Fig. 4D

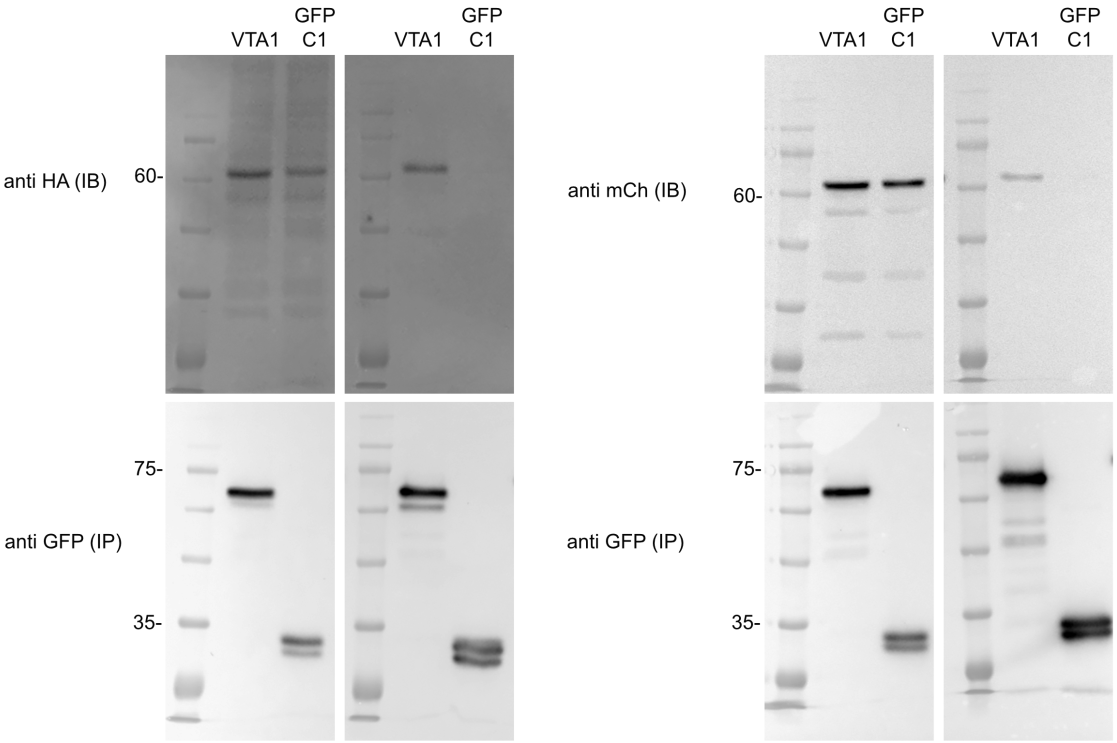

Complete blots Fig. 4E

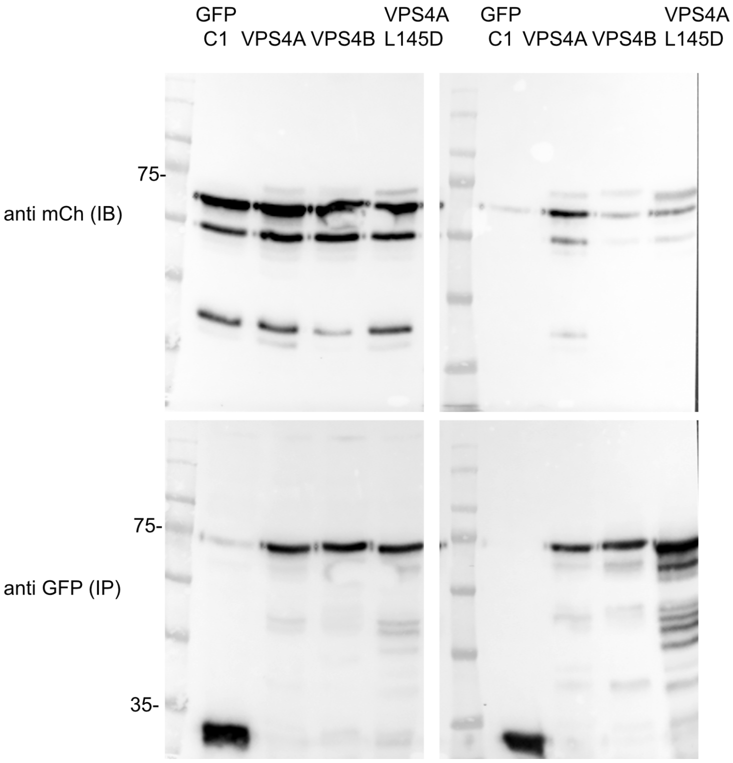

Complete blots Fig. 5A

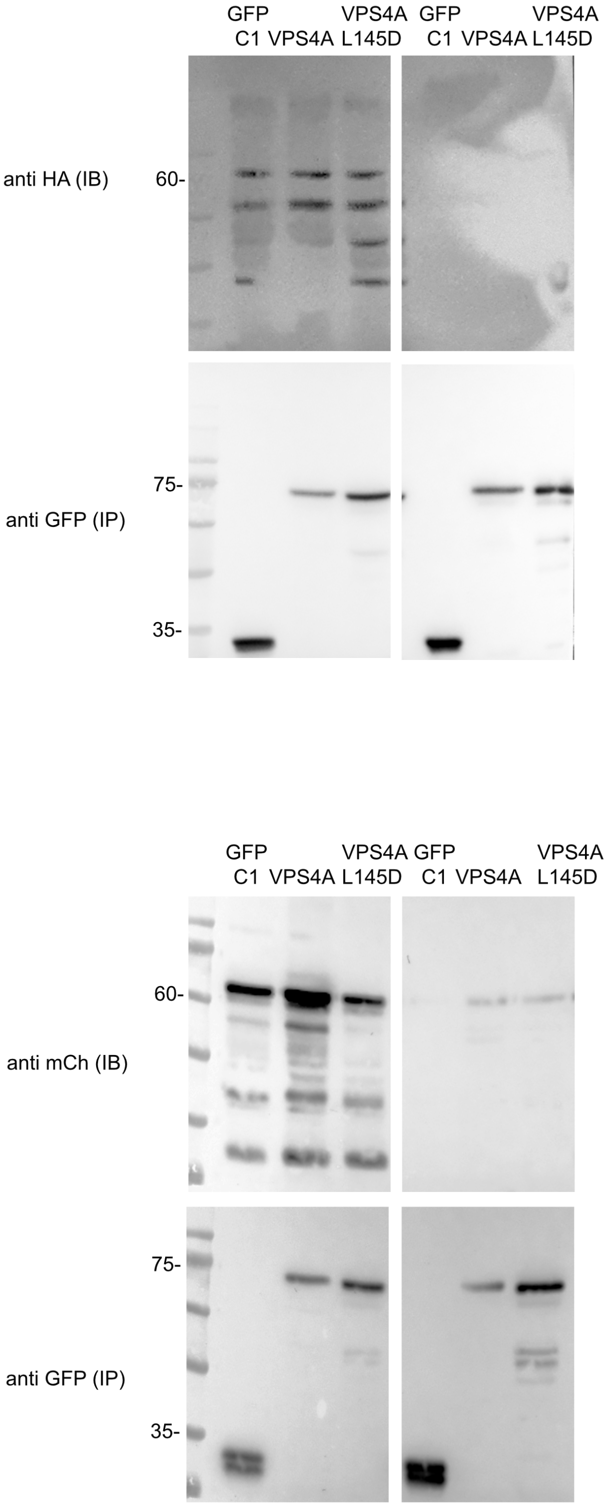

Complete blots Fig. 5B

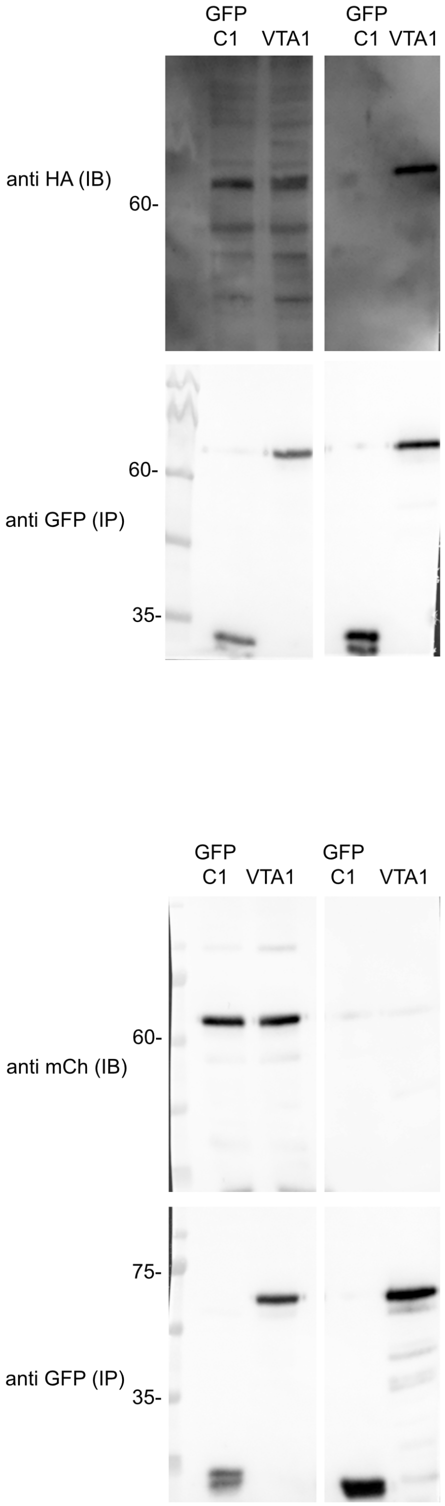

Supplement: S1 Raw Images — (PDF) [file pbio.3002327.s011.pdf]
